# Phylogeny with introgression in *Habronattus* jumping spiders (Araneae: Salticidae)

**DOI:** 10.1186/s12862-018-1137-x

**Published:** 2018-02-22

**Authors:** Geneviève Leduc-Robert, Wayne P. Maddison

**Affiliations:** 1 Department of Zoology, University of British Columbia, Vancouver, BC V6T 1Z4 Canada; 2 Department of Botany and Beaty Biodiversity Museum, University of British Columbia, Vancouver, BC V6T 1Z4 Canada

**Keywords:** Phylogeny, Introgression, Hybridization, Transcriptome, Jumping spiders, Salticidae, Salticinae, Plexippini, Harmochirina, *Habronattus*

## Abstract

**Background:**

*Habronattus* is a diverse clade of jumping spiders with complex courtship displays and repeated evolution of Y chromosomes. A well-resolved species phylogeny would provide an important framework to study these traits, but has not yet been achieved, in part because the few genes available in past studies gave conflicting signals. Such discordant gene trees could be the result of incomplete lineage sorting (ILS) in recently diverged parts of the phylogeny, but there are indications that introgression could be a source of conflict.

**Results:**

To infer *Habronattus* phylogeny and investigate the cause of gene tree discordance, we assembled transcriptomes for 34 *Habronattus* species and 2 outgroups. The concatenated 2.41 Mb of nuclear data (1877 loci) resolved phylogeny by Maximum Likelihood (ML) with high bootstrap support (95-100%) at most nodes, with some uncertainty surrounding the relationships of *H. icenoglei*, *H. cambridgei, H. oregonensis,* and *Pellenes canadensis*. Species tree analyses by ASTRAL and SVDQuartets gave almost completely congruent results. Several nodes in the ML phylogeny from 12.33 kb of mitochondrial data are incongruent with the nuclear phylogeny and indicate possible mitochondrial introgression: the internal relationships of the *americanus* and the *coecatus* groups, the relationship between the *altanus, decorus, banksi*, and *americanus* group, and between *H. clypeatus* and the *coecatus* group. To determine the relative contributions of ILS and introgression, we analyzed gene tree discordance for nuclear loci longer than 1 kb using Bayesian Concordance Analysis (BCA) for the *americanus* group (679 loci) and the VCCR clade (*viridipes/clypeatus/coecatus/roberti* groups) (517 loci) and found signals of introgression in both. Finally, we tested specifically for introgression in the concatenated nuclear matrix with Patterson’s D statistics and D_FOIL_. We found nuclear introgression resulting in substantial admixture between *americanus* group species*,* between *H. roberti* and the *clypeatus* group, and between the *clypeatus* and *coecatus* groups.

**Conclusions:**

Our results indicate that the phylogenetic history of *Habronattus* is predominantly a diverging tree, but that hybridization may have been common between phylogenetically distant species, especially in subgroups with complex courtship displays.

**Electronic supplementary material:**

The online version of this article (10.1186/s12862-018-1137-x) contains supplementary material, which is available to authorized users.

## Background

The visually-acute jumping spiders include *Habronattus*, a clade of about 100 described species whose colourful and diverse courtship displays are among the most complex found in arthropods [1–6]. The group is emerging as a model to study the role of sexual selection in divergence [4, 7–11], the evolution of sex chromosomes [12, 13], and arthropod visual systems [14–16].

Although these studies of characters and diversification in *Habronattus* have been guided by our knowledge of the group’s phylogeny, their ability to clearly trace evolutionary processes in this densely diverse group has been limited by poor phylogenetic resolution. Several subclades were recognized by distinctive structures and behaviours by Griswold [3] and confirmed by molecular data from two gene regions (nuclear Ef1-α and mitochondrial 16S-ND1, [5]). These include a large clade of 42 species whose males have fringed first legs and modified third legs (here called the VCCR clade, subdivided into the *viridipes*, *coecatus*, *clypeatus*, and *roberti* groups), the *americanus* group, the *dorotheae* group, and several groups of robust-bodied, often shrub-dwelling species (*agilis*, *amicus*, *tranquillus* groups, collectively named here the AAT clade). Other well-supported groups, such as the *decorus* group, emerged only with molecular data [5]. However, relationships within and among these species groups are, for the most part, little resolved (see, e.g., the conservative tree of Figure 4 in [12]).

Difficulties in resolving *Habronattus* phylogeny may stem from the recency of its diversification — possibly less than 5 million years (Figure 8 in [17]) — yielding insufficient sequence divergence or high rates of incomplete lineage sorting (ILS, [18]). Maddison and Hedin’s [5] gene trees show several cases of distinct morphospecies intermingling when represented by multiple specimens (e.g., *H. pyrrithrix*, *H. virgulatus*, and other *coecatus* group members). In addition, the recency of their divergence may leave *Habronattus* species susceptible to hybridization [19, 20], leading to introgression and thus discordant phylogenetic signals.

Indeed, signals of introgression have been noted in mitochondrial data between sympatric and closely related species [21] and even between distant species whose courtship ornaments and genitalia differ markedly [5]. Hints are also seen in sexually selected ornaments in males from divergent *H. pugillis* populations, whose patterns of convergence could be explained by introgression [4]. Hybrid zones are known among several pairs of closely-related species ([36], unpublished observations). The possibility of introgression is supported by behavioural studies, which have found that females from different populations of *H. pugillis* have preferences for foreign males with divergent courtship displays, a possible result of antagonistic coevolution between the sexes [7]. If this has happened throughout *Habronattus*, then we may be faced with an unfortunate irony: the very processes of sexual selection that make this group so tempting to study may at the same time obscure the phylogenetic history we depend on for comparative analyses.

To whatever extent a divergent phylogeny exists in the group, our goal here is to use genomic data to resolve it. We also seek to determine whether the previously-inferred mitochondrial introgression in *Habronattus* stands alone as in other taxa (e.g., [23]) or is accompanied by nuclear introgression. Such introgression could do more than confuse phylogeneticists; it could have introduced new genetic variation at a rate faster than possible by mutation alone, leading to the sharing of adaptive loci across lineages and facilitating diversification [24–26]. Although introgression of courtship traits into established systems of mate choice with such elaborate signals might seem difficult, sensory bias, Fisherian runaway, and antagonistic coevolution models could all promote this dynamic [7, 9, 27, 28]. Determining how much genetic discordance in *Habronattus* can be attributed to introgression may provide crucial insights into whether hybridization may have had any substantial creative role in the group’s diversification.

The importance of introgression in animal evolution is uncertain [29], in part because distinguishing discordant signals resulting from ILS and introgression is difficult without phylogenomic datasets [30–33]. We collected transcriptome data for 34 *Habronattus* species and two outgroups, the first genomic dataset assembled for salticid spiders. With a well-resolved phylogenetic tree, we were then able to use a phylogenetic approach to characterize nuclear and mitochondrial introgression in the group. We focused on the *americanus* group and the VCCR clade, two of the most diverse clades within *Habronattus*. To investigate nuclear introgression, we first conducted a Bayesian Concordance Analysis [33, 34] to investigate discordance (caused by either ILS and/or introgression) in gene trees, and we then applied Patterson’s D statistic [32] and D_FOIL_ [22] tests to explicitly test allele patterns for introgression. We were able to resolve most nodes of the phylogeny with high support and identified several instances of hybridization in the group.

## Methods

### Taxon sampling

We sampled a total of 36 species, including representatives from most major clades within *Habronattus,* and 2 outgroups (Table 1). We sampled more extensively in species-rich groups (i.e., *viridipes/clypeatus/coecatus* group, and the *americanus* group), and prioritized the *coecatus* group because of possible introgression [5]. This is the first phylogenetic analysis to include the species *H. aestus, H. empyrus,* and *H. luminosus* [35]. Some of our sampled species were studied by Maddison and Hedin [5] under different names (*H. chamela*, *H. gilaensis*, *H. roberti*; see [35, 36]). The outgroup *Pellenes canadensis* is closely related to *Habronattus* within the subtribe Harmochirina*,* while *Evarcha proszynskii* is more distantly related, in the neighboring subtribe Plexippina [17, 37].

**Table 1:** Specimens of *Habronattus* and outgroups sequenced

Species	Voucher		Locality, with latitude and longitude	
Outgroups				
*Evarcha proszynskii* Marusik & Logunov, 1998	GLR135	♂	Canada: BC: Mission	49.166, − 122.409
*Pellenes canadensis* Maddison 017	GLR106	♂	Canada: BC: Mt. Baldy Road	49.1135, − 119.2103
AAT Clade				
*Habronattus conjunctus* (Banks, 1898)	GLR234	♂	U.S.A.: AZ: Madera Canyon	31.7417, −110.8847
*H. hirsutus* (Peckham & Peckham, 1888)	GLR080	♂	Canada: BC: Mt. Kobau Road	49.095, −119.610
*H. signatus* (Banks, 1900)	–	–	U.S.A.: CA: Ocotillo	32.7421, −115.9949
*H. ustulatus* (Griswold, 1979)	–	–	U.S.A.: CA: Boulder Oaks	32.7302, −116.4607
*americanus* group				
*H. aestus* Maddison 2017	GLR287	♀	México: Sonora: Puerto Peñasco	31.418, −113.626
*H. americanus* (Keyserling, 1885)	GLR014	♂	Canada: BC: Iona Beach	49.221, −123.214
*H. ophrys* Griswold, 1987	GLR023	♂	Canada: BC: Iona Beach	49.221, −123.214
*H. ophrys*	GLR015	♀	Canada: BC: Iona Beach	49.221, −123.214
*H. sansoni* (Emerton, 1915)	GLR066	♂	Canada: BC: Kelowna	49.954, −119.398
*H. tarsalis* (Banks, 1904)	GLR297	♂	U.S.A.: AZ: Yuma	32.731, − 114.612
DTB clade				
*H. altanus* (Gertsch, 1934)	GLR180	♂	Canada: AB: Smoky Lake	54.112, −112.198
*H. chamela* Maddison 2017	GLR352	♂	México: Jalisco: Chamela	19.5038, −105.0334
*H. decorus* (Blackwall, 1846)	GLR132	♂	Canada: BC: Mission	49.166, −122.409
*H. zapotecanus* Griswold, 1987	GLR339	♂	México: Jalisco: Chamela	19.5316, −105.0707
*roberti* group				
*H. roberti* Maddison 2017	JAL14-9281	♂	México: Jalisco: Chamela	19.496, −105.042
*viridipes* group				
*H. calcaratus maddisoni* Griswold, 1987	GLR321	♂	Canada: ON: Haileybury	47.45, −79.708
*H. jucundus* (Peckham & Peckham, 1909)	GLR320	♂	U.S.A.: OR: Bolan Lake,	42.024, −123.461
*clypeatus* group				
*H. aztecanus* (Banks, 1898)	GLR347	♂	México: Jalisco: Puerto Vallarta	20.670, −105.274
*H. clypeatus* (Banks, 1895)	GLR227	♂	U.S.A.: AZ: Mt. Hopkins Road	31.686, −110.975
*H. gilaensis* Maddison & Maddison 2016	AS56	♀	U.S.A.: New Mexico: Silver City	
*coecatus* group				
*H. borealis* (Banks, 1895)	GLR040	♂	Canada: ON: Burlington	43.33, −79.8
*H. captiosus* (Gertsch, 1934)	GLR356	♀	Canada: AB: Guy	55.4505, −117.1440
*H. empyrus* Maddison 2017	GLR282	♂	México: Sonora: Puerto Peñasco	31.293, −113.452
*H. festus* (Peckham & Peckham, 1901)	GLR094	♂	Canada: BC: Hayne’s Lease	49.0813, −119.5181
*H. festus*	GLR088	♀	Canada: BC: Hayne’s Lease	49.0813, −119.5181
*H. mexicanus* (Peckham & Peckham, 1896)	GLR353	♂	México: Jalisco: El Tuito	20.341, −105.350
*H. pyrrithrix* (Chamberlin, 1924)	GLR304	♂	U.S.A.: AZ: Yuma	32.731, − 114.612
*H. virgulatus* Griswold, 1987	GLR205	♂	U.S.A.: AZ: Mt. Hopkins Road	31.689, −110.975
Other *Habronattus*				
*H. cambridgei* Bryant, 1948	GLR351	♂	México: Jalisco: Puerto Vallarta	20.670, −105.274
*H. geronimoi* Griswold, 1987	GLR267	♂	U.S.A.: AZ: Miller Canyon	31.416, −110.276
*H. hallani* (Richman, 1973)	GLR209	♂	U.S.A.: AZ: Arivaca	31.668, −111.245
*H. icenoglei* (Griswold, 1979)	GLR283	♂	México: Sonora: Puerto Peñasco	31.273, −113.361
*H. luminosus* Maddison 2017	GLR218	♀	U.S.A.: AZ: Mt. Hopkins Road	31.6759, −110.9289
*H. oregonensis* (Peckham & Peckham, 1888)	GLR149	♂	Canada: BC: Squamish	49.8465, −123.1452
*H. paratus* (Peckham & Peckham, 1896)	GLR363	♂	Panama: Isla Colon	9.40376, −79.8635
*H. pugillis* Griswold, 1987	GLR236	♂	U.S.A.: AZ: Mt. Hopkins Road	31.689, −110.975

Specimens were collected from 2012 to 2014 from the locations listed in Table 1, following institutional and governmental regulations. Permits for specimens from Mexico were granted through the collaboration of Dr. Tila María Perez by the Secretaría de Medio Ambiente y Recursos Naturales (Semarnat), Mexico. Adult male specimens were preferred because they are easier to identify morphologically to species. We resorted to adult females for *H. aestus, H. luminosus, H. captiosus,* and *H. gilaensis* because males were not available, but in each case there are no sympatric closely related species likely to be confused with them, and males have been collected from the same locations. Both a male and a female specimen were included for *H. ophrys* and *H. festus* in an effort to assemble a more complete reference transcriptome.

DNA of *H. paratus* was preserved in 95% EtOH. All other specimens were killed by submersion in RNAlater for RNA preservation. To maximize tissue exposure, the cephalothorax and abdomen were opened immediately upon submersion. All specimens were stored at − 20 °C. Legs and the male palps or the female epigynum were preserved separately as vouchers (stored at the Beaty Biodiversity Museum at the University of British Columbia, Table 1).

### Molecular extractions and sequencing

Total RNA was extracted from whole specimens using a combination of TRIzol extraction (Life Technologies) and RNeasy Mini Kit (Qiagen) for RNA purification and DNAse digestion. DNA was extracted from the legs and abdomen of *H. paratus* using a QIAamp DNA Investigator Kit (Qiagen), assessed for integrity on a 21,000 Bioanalyzer. Libraries were constructed with BIO-O NEXTflex Library Prep Kits (Bioo Scientific, Inc.) with insert sizes averaging 220 bp for RNA and 300 bp for DNA, and sequenced as 100 bp paired-end reads on an Illumina HiSeq 2000 (Illumina, Inc.) at the Biodiversity Research Centre Next Generation Sequencing Facilities (University of British Columbia). For economic feasibility we chose a strategy of building a de novo assembled transcriptome for one of two species that were deeply sequenced (*H. ophrys* and *H. festus*), then assembling the other species by reference to it, allowing them to be less deeply sequenced. For the latter species, we aimed for at least 20 million paired reads per species before trimming, and achieved 5-30 million reads after trimming (see Additional file 1: Table S1 for sequencing summary). Marshal Hedin supplied sequence reads of transcriptomes of *H. signatus* and *H. ustulatus,* prepared and sequenced as 50 bp paired end sequences, and Megan Porter supplied sequence reads of *H. gilaensis*, prepared and sequenced as 150 bp paired end sequences. Sequence reads are deposited in the Sequence Read Archive (SRA submission SUB3319693 [38]); accession numbers are in Additional file 1: Table S1.

### Sequence read filtering and trimming

Any sequence read with an average Phred score under Q = 30 was discarded. All remaining reads were quality checked with FASTQC V0.10.1 [39]. Terminal nucleotides were trimmed using fastq-mcf from ea-utils [40] if they had a score below Q = 30 or if they were sequencing adaptors. Reads that were 95% or more homopolymer were discarded and any suspected contaminants, detected from the GC content curve of FASTQC, were trimmed using PRINSEQ-lite [41]. Any read shorter than 33 bp after trimming was discarded.

### Reference transcriptome

Transcriptomes were assembled de novo for *H. festus* and *H. ophrys* in Trinity RNA-seq_v20140717 [42] with the command: “Trinity.pl –seqType fq –left leftreads.fq –right rightreads.fq --JM 110G –CPU 12 –inchworm_cpu 12 –bflyCPU 12 –min_contig_length 200 –-kmer_cov 2”.

Both assemblies were similar in size and quality, so we selected *H. ophrys* as the reference transcriptome because of predicted ease of obtaining this species for future studies.

We filtered the approximately 100,000 transcripts from *H. ophrys* prior to using its transcriptome as a reference for subsequent assemblies. We determined transcript abundance with RSEM v1.2.19 [43] and kept only the most abundant transcript variant per gene. Any remaining redundant transcripts identified with CD-HIT-EST [44] with a similarity threshold of 95% were removed (176 transcripts removed). To decrease the likelihood of paralogous genes assembling on a reference transcript during reference-based assemblies, we conducted an all-versus-all BLAST with all remaining *H. ophrys* contigs and removed any contig with a contig other than itself as a significant hit (blastx, evalue = 10^− 3^; 34 transcripts removed). To set codon positions, the reference transcriptome was scanned for open reading frames using TransDecoder_r20131110 [42] and the longest open reading frame of a transcript was chosen as its protein coding region. If multiple non-overlapping coding regions were found on a transcript, those transcripts were split between coding regions.

We conducted a BLAST search of the entire *H. ophrys* transcriptome (evalue = 10^− 3^, min. HSP length = 33, max_target_seqs = 20) to the *H. oregonensis* mitochondrial genome [45] and any significant hits were removed from the final reference nuclear transcriptome. The annotated *H. oregonensis* mitochondrial genome [45], was used instead as the reference for all mitochondrial assemblies.

### Reference-based assembly of transcriptomes

For every species (including reference species *H.ophrys)*, sequencing reads were first mapped to the *H. oregonensis* mitochondrial genome and the remaining unmapped reads were mapped to the reference transcriptome using CLC Genomics Workbench (CLC Bio), chosen because of its extensive facilities for making and visualizing reference-based assemblies. Reads of *H. ophrys* were also remapped to its own reference so as to obtain sequencing depth information and follow a trimming protocol comparable to the other species. Assembly parameters used were: mismatch cost = 2, insertion cost = 3, deletion cost = 3, length fraction = 0.5, similarity fraction = 0.8. When consensus sequences were extracted from the read mappings, polymorphisms were retained as ambiguity codes in the consensus sequence if the variant represented 30% or more of mapped alleles, except for *H. festus* which used a combination of read count and sum of quality scores to resolve the base conflict. Nuclear sequences with average sequencing depth less than 5× were discarded, and contigs were split into fragments at any region where sequencing depth was less than 5×. Following these steps, only contigs longer than 200 bp were retained. Total raw sequencing reads, trimmed sequencing reads, the number of reads assembled, total transcripts, and sequencing depth are summarized for every species in Additional file 1: Table S1. Only mitochondrial data were used for *H. paratus*, as most of its nuclear sequences were incomplete or with inadequate sequencing depth.

### Alignment and filtering of loci

We converted species-based FASTA files into locus-based FASTA files and trimmed sequences of any remaining poly-A or poly-T tails using custom python scripts. If a locus was fragmented into multiple sequences for a species, and the lengths of those fragments when added together met our length cut-off (200 bp), then those sequence fragments were scaffolded using their positions aligned against the reference transcript, filling in with “N”s to represent the missing data.

At this stage, there were 55,316 loci, most present only in the two species with a much higher number of reads, *H. ophrys* and *H. festus*. Of those, 7132 were present in at least 15 species, and 4188 were present in at least 25 species. Each locus was aligned using MAFFT v7.058b [46] using L-INS-I and parameters –localpair –maxiterate 100. Alignments of a few loci were manually corrected to resolve obvious reading frame misalignments. Nucleotides trailing from either end of an alignment (present in 30% or less of species) were trimmed and a sequence was discarded if it was 30% or less than the average sequence length for that alignment. All alignment, trimming and partitioning steps were completed with the aid of Mesquite 3.02 [47], with a few steps involving the package Gataga (Maddison and Maddison, unpubl.).

The aligned matrix for each locus was partitioned by codon position or non-coding based on Transdecoder results. To better understand the cause of gaps and ambiguous nucleotides in these alignments, we aligned a subset of loci with annotated sequences with known protein-coding regions matched using a BLAST search to the SWISS-PROT database, to Ef1-α [5], and to the annotated mitochondrial genome [45]. All sites with gaps were caused by an insertion of a nucleotide in one or a few sequences, implying highly unlikely frame-shifts in conserved genes. These insertions occurred only in the reference-based assemblies and never occurred in the (higher quality) reference sequence. Therefore, we inferred that these insertions of gaps and nucleotides were assembly errors rather than true insertions. As a result, columns with gaps were excluded whenever coding-region gaps were not present in the reference sequence (to avoid any frameshift mutations). In noncoding regions there were also occasionally insertions in some species — in the vast majority of columns, in only one or two species, again suggesting assembly errors. However, a more relaxed exclusion criterion was used for non-coding regions: a column was excluded if the insertion was present in less than 50% of species.

Some loci showed high levels of ambiguous sites, which might not represent true heterozygosity, but rather multiple transcript variants or paralogs assembled on the same reference transcript. To be conservative, loci were excluded from analyses if ambiguous sites constituted more than 3% of the alignment. 236 loci were thereby removed, resulting in 1877 loci in our highest quality subset (present in at least 25 species, at least 200 bp long, with a coding region identified by Transdecoder, and with less than 3% ambiguity).

Because reference-based assemblies generate sequences aligned to the same reference transcripts, sequences assembled on the same reference are treated as orthologous. Filtering for paralogs in the *H. ophrys* reference transcriptome, reducing reference transcriptome redundancy, and removing sequences with high levels of ambiguous sites are expected to have reduced the possibility of paralogs. To assess whether there were obvious paralogs, gene trees were constructed for all genes of alignment lengths over 1 kb with a single search replicate in RAxML 7.7.9 [48]. None of the trees produced had unusually long branches or phylogenetic structures obviously indicative of paralogy. While this reassures us that there were not deep paralogs, some more recent paralogs may have passed through our filters. Our expectation is, however, that the effect of paralogs averaged over many loci would be to add noise rather than systematic biases, as noted in the discussion about introgression and artifacts.

#### Nuclear subsets

We separated the nuclear loci into four groups. The first group, the Primary Subset, included the 1877 highest quality loci as described above (2.41 Mb alignment; average of 2,036,173 base pairs per species). These were used for our primary nuclear phylogenetic analyses. Among the remnant “low quality” loci, those matching the criteria of high quality except for being present in only 15 to 24 species were treated as the second group, the Missing Species (MS) subset (1019 loci; average of 548,107 bp per species). The other remnants without an identifiable coding region but present in 25 or more species were the third group, the Noncoding Loci (NL) subset (236 loci; average of 92,567 bp per species). The fourth group consisted of loci not meeting any of these criteria; they were discarded.

#### Mitochondrial data

To compose a complete mitochondrial alignment, sequences for 16S RNA (1022 bp), 12S RNA (691 bp), ND1 (921 bp), ND2 (959 bp), ND3 (342 bp), ND4 (1289 bp), ND4L (268 bp), ND5 (1638 bp), ND6 (429 bp), ATP6 (666 bp), ATP8 (158 bp), Cytochrome B (1111 bp), COX1 (1542 bp), COX2 (666 bp), COX3 (786 bp) were aligned, concatenated, and assigned codon positions based on annotations from the *H. oregonensis* mitochondrial genome [45]. This yielded the concatenated mitochondrial matrix, with a total alignment length of 12.33 kb.

Maddison and Hedin’s [5] two-gene *Habronattus* phylogeny includes many species absent in our transcriptome data. In order to get a denser perspective on mitochondrial introgression within the VCCR clade, we took sequences from the 16SND1 region from our mitochondrial data (1047 bp) for our 35 transcriptome species and DNA specimen *H. paratus*, and added to them data for the same region obtained by Maddison and Hedin [5], Masta and Boore [45], and Masta and Maddison [11]. Combined these yielded a matrix with 196 sequences of 16SND1 from across the genus, including 37 VCCR species (in comparison to 13 species in our transcriptome data).

### Phylogenetic analyses

*Habronattus* species phylogeny from nuclear genes was inferred by maximum likelihood from concatenated alignments, as well as by coalescent-based methods (ASTRAL, SVDQuartets).

#### Maximum likelihood on concatenated matrices

All maximum likelihood phylogenetic searches on the concatenated matrices were run in RAxML 7.7.9 [48] with 20 search replicates for the ML tree, and one search replicate for each of the 1000 bootstrap replicates. We used PartitionFinder (v.1.1.1 [49]) to assess the substitution models, using a greedy algorithm search and AIC model selection, and considering both locus and codon positions as possible partitioning criteria.

In addition to the primary concatenated matrix of 1877 genes, 12 other matrices were analyzed representing different subsets of nuclear loci, to explore the consistency of support. Eight of these were equal subsets of our primary matrix (nuclear subsets 1-8; 302,200 bp each). Locus order was randomized prior to subset division and concatenation to ensure that each subset represents a random sample of loci. In addition, the remnant (low quality) loci were used to make two other disjoint matrices (MS and NL, as defined above).

To assess the strength of the mitochondrial phylogenetic signal with less data, we divided mitochondrial rRNA (1.72 kb) from protein-coding sites and then separated the protein-coding alignment into 4 even subsets (mtDNA subsets 1-4; 2.53 kb each).

In the analysis for the expanded 16SND1 matrix we constrained the inference to enforce any node in the concatenated mitochondrial tree that had at least 90% bootstrap support. We did this so as to take advantage of the strong resolution available for the 36 transcriptome taxa, and to determine how the additional sequences fell on that skeletal constraint tree. Although we performed the analysis across the whole genus, our results focus on the VCCR clade.

#### Coalescent methods for the species tree

Using the primary subset of nuclear genes (1877 loci), two methods based on multi-species coalescent models were used to infer the species phylogeny, ASTRAL [50] and SVDQuartets [51]. ASTRAL version 4.7.12 [52] was applied to the 1020 loci from the primary nuclear subset of alignment length at least 1000 bp. For each locus, a single gene tree was reconstructed by a simple maximum likelihood search by RAxML (model GTRGAMMAI, unpartitioned), and the set of 1020 gene trees was analyzed by ASTRAL using default settings. For the SVDQuartets analysis, PAUP* version 4.0a149 (2016; [53]) was applied to the primary concatenated nuclear matrix (2.41 Mb alignment), default search settings, with 1000 bootstrap replicates.

### Analysis of introgression

To assess signals of introgression in the nuclear data, we examined nuclear signals discordant with the species tree using Bayesian Concordance Analysis (BCA), and tested for introgression using D-statistics.

#### Bayesian concordance analysis

We used BCA to assess the extent of nuclear discordance and the possibility of introgression [33, 34]. From a Bayesian sample of gene trees for each locus, BCA derives a primary concordance tree representing the dominant phylogenetic signal (an estimate of the species tree). and secondary concordance factors (CF) indicating substantial support from some genes but discordant with the dominant species tree [33, 34, 54]. While BCA does not test explicitly for introgression, if two conflicting secondary CFs are unequal and their 95% credibility intervals do not overlap (and therefore are significantly different), introgression may be inferred as a possible cause of discordance because under ILS the CFs for conflicting secondary clades would be expected to be equal [54].

We conducted all Bayesian gene tree searches with MrBayes 3.2.2 [55] with 4 chains (3 cold, 1 hot), 2 runs, and 25% burn-in. To determine the number of MCMC generations required for convergence, 20 genes were run until convergence (standard deviation of split frequencies < 0.01; [56]) as a test. The convergence time ranged from 570,000 to 13,087,000 and averaged 4,025,500 generations. Based on this, we set the number MCMC generations conservatively at 20,000,000 generations per gene. Codon positions were used as partitions.

BCA analyses were conducted using BUCKy 1.4.3 [57] with 2 runs and 4 chains per analysis. Due to computational limitations, we analyzed only the *americanus* group and the VCCR clade, and included only genes longer than 1 kb that were present in all species being analyzed. There were 679 genes included for the *americanus* group analysis (with *H. signatus* as outgroup) and 517 genes included for the VCCR clade analysis (with *H. ophrys* as outgroup).

For the adjustable prior α, the a priori level of gene tree incongruence, we tried values of α = 0.1, 1, 2, 5 and 10. We found no substantial difference in results using different α for the *americanus* group, so kept α set to 1. Analyses involving the VCCR clade had difficulty converging at higher α, so α was set to 0.1 for that analysis, although there was very little difference between CFs depending on α for this clade. The *americanus* group analysis ran for 10,000,000 generations and the VCCR clade analysis ran for 30,000,000 generations due to the longer time required for convergence.

#### Patterson’s D statistic and D_FOIL_

To distinguish incomplete lineage sorting (ILS) and introgression patterns in SNP data, we conducted Patterson’s D statistic tests [58] and the related test D_FOIL_ [22]*.* These tests compare patterns of shared SNPs across sets of 4 and 5 taxa, respectively. Under ILS in a 4-taxa binary tree, it is expected that the number of alleles shared by non-sister taxa (i.e. discordant with the species tree) would be equal for each possible non-sister pairing (i.e., patterns ABBA and BABA) [32]. Introgression between two species is inferred when they have significantly more shared alleles than alternative discordant pairs (i.e., more ABBA than BABA or vice versa). The same principle can be applied to a 5-taxa tree with structure (((species 1, species 2),(species 3, species 4)), outgroup), with some added complexity [22]. Whether Patterson’s D statistic or D_FOIL_ was used depended on the structure of the species tree of the taxa being tested.

We tested sets of species based on indications of introgression in previous studies [5], in the mitochondrial phylogeny, or in the BCA. There were 3 principal hypotheses of introgression tested: (1) among species of the *americanus* group, (2) between *H. roberti* and the other groups of the VCCR clade and (3) between the *clypeatus* group and *coecatus* group. In addition, the possibility of very distant introgression between the DTB clade (*decorus*/*texanus*/*banksi* groups), VCCR clade and the *americanus* group was also explored.

For the *americanus* group tests, we included all 5 *americanus* group species. For the other tests, we selected a few species as representatives of each clade. For the *H. roberti*/VCCR D statistics analysis, we used the species with the most sequencing data per clade for the four clades involved (*H. roberti*, and one representative of each of the *viridipes*, *clypeatus, coecatus* groups). For the *clypeatus* group - *coecatus* group analysis, we did 6 combinations of species, including 2 *coecatus* group and 2 *clypeatus* group species per D_FOIL_ test, and using *H. ophrys* as the fifth species (outgroup). *H. pyrrithrix* (*coecatus* group) was included in each combination because its phylogenetic position is consistent in both the concatenated nuclear and mitochondrial phylogenies; *H. clypeatus* (*clypeatus* group) was included because of mitochondrial introgression detected in the mitochondrial phylogeny and in other members of its species group by Maddison and Hedin [5]. By using more or less divergent sister taxa in different tests for comparison, we can better approximate where in the *coecatus* and *clypeatus* group introgression occurred. Even still, D-statistics and D_FOIL_ cannot rule out the possibility that detected introgression was with related lineages that are extinct or were otherwise not sampled (i.e., ghost lineages) [59].

D_FOIL_ was run in mode dfoilalt to reduce noise from synapomorphic sites. We used a custom R script to count allele patterns. All sites that included ambiguous nucleotides, gaps, or missing data were excluded from the analysis. D_FOIL_ estimated divergence T-values were verified against the assumption that T_12_ < T_34_ < T_1234_. Because each D_FOIL_ and D-test is a chi-square binomial test, we adjusted significance for multiple comparisons (62 in total, including all D_FOIL_ and D tests) with a Bonferroni correction to a *p*-value lower than 0.0008 to indicate 95% significance.

## Results

### Transcriptome assemblies and data filtering

The unfiltered *H. ophrys* reference transcriptome included 117,859 transcripts (total 53,927,457 bases assembled), with an N50 (analogous to median contig length; [60]) of 516, and an average sequencing depth of 103×. Following filtering for redundancy, selection of a single variant per gene, removal of possible paralogs, and the separation of connected transcripts, there were 92,343 transcripts left.

Additional file 1: Table S1 gives a summary of transcriptome assemblies, excluding unused sequences with low (< 5×) sequencing depth. After reads were remapped, 51,143 *H.ophrys* transcripts had sufficient (5×) sequencing depth (average sequencing depth 111×). For all other species, reference-based transcriptome assemblies mapped on average 77% of trimmed reads to either the nuclear reference transcriptome (average nuclear sequencing depth = 67×) or mitochondrial reference genome (average mitochondrial sequencing depth = 13,640×). There was an average of 10,164 transcripts assembled per species, although numbers ranged widely (depending on the number of reads) from 3746 for *H. roberti* to 28,846 for *H. festus*. Aligned matrices for each of the partitions (Primary, Missing Species, Noncoding Loci, and mitochondrial) are available in Additional file 2.

### Substitution model selection

For the primary concatenated nuclear matrix, PartitionFinder was unable to analyze the approximately 7500 partitions (codon positions for each of 1877 genes) because of computational limits. Thus, we applied it to the mitochondrial genes and on a sample of 20 nuclear genes, using it to assess models rather than choose partitions. GTR + G + I or GTR + G was chosen as the optimal substitution model using AIC for all mitochondrial partitions. For the 20 nuclear genes tested, 33 partitions had a GTR model selected, 22 had TVM, 10 had K81uf, 5 had TIM, 4 had HKY, and 3 had TrN (these number include model variations like +G or +I). We were unable to set a different model for each partition in concatenated matrices due to computational limitations. Instead, we set GTR + G + I as the substitution model in all maximum likelihood (ML) and Bayesian analyses (nst = 6 rates = invgamma) because it was the most commonly chosen and most widely applicable mode. We used 4 partitions based on codon position (position 1, position 2, position 3, and noncoding) for all figured analyses. To test whether partitioning played a major role, we also ran an unpartitioned likelihood analysis on the 1877 locus concatenated nuclear matrix, as well as one partitioned by locus.

### Nuclear phylogeny

**Fig. 1:**
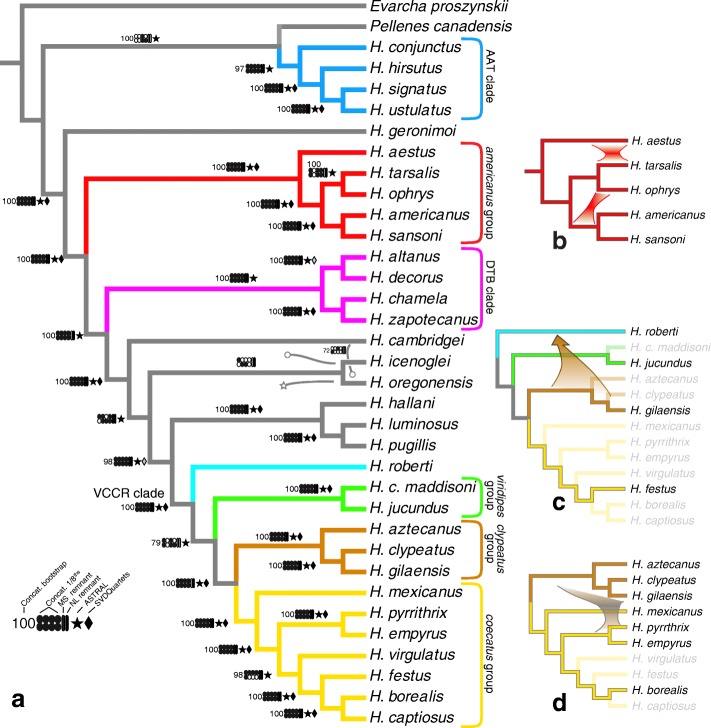
Species phylogeny from nuclear data in *Habronattus* (for branch lengths, see Fig. 2a), and main conclusions of nuclear introgression. **a** Maximum likelihood tree from 1877 concatenated nuclear loci (2.41mb alignment; RAxML); largely concordant with the ASTRAL tree (stars). Colours mark species groups as labeled. Legend for decorations at lower left. Bootstrap percentages for ML analysis of concatenated nuclear matrix. Spots and vertical bars show presence of clade in the ML tree for various partitions of the data (black = clade present). Spots show presence of clade in tree for each of the 1/8th portions of concatenated nuclear loci. Vertical bars show presence of clade in the trees from the remnant Missing Species (MS) and the Noncoding Loci (NL) matrices. Stars show clades present in ASTRAL analysis of 1020 ML gene trees of alignment length at least 1000 bp. Diamonds show clades with (black) > 94% or (grey) 75-94% bootstrap support in SVDQuartets analysis. Discordant results shown by grey arrows with circle (concatenated ML bootstrap consensus) or star (ASTRAL). For instance, the concatenated bootstrap consensus places *H. cambridgei* as sister to *oregonensis*, and *H. icenoglei* in a more basal position with respect to the VCCR clade. From **b** to **d**: Conclusions of introgression from D-statistics and BCA for **b** the *americanus* group, **c** *H. roberti* and the VCCR clade, and **d** the *clypeatus* and *coecatus* groups. See text for signals of other introgression events

**Fig. 2:**
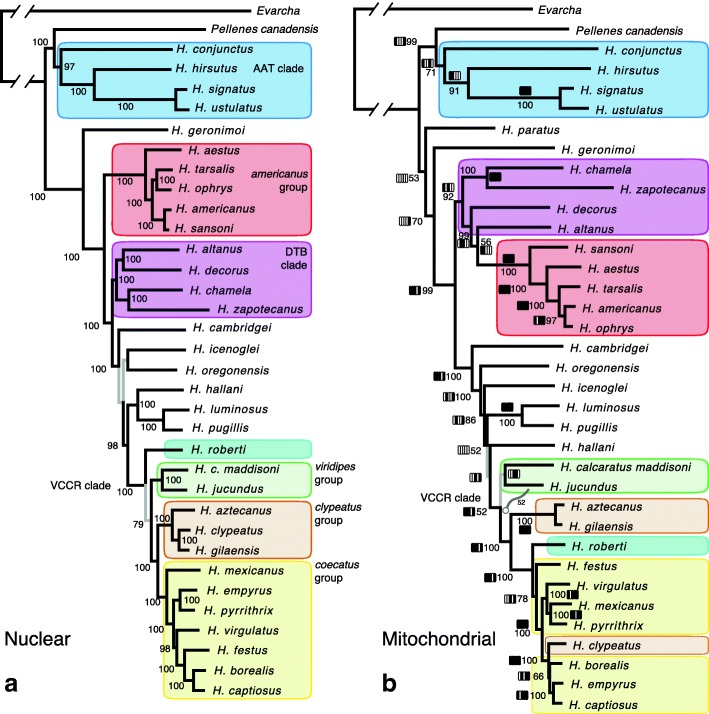
Comparison of nuclear and mitochondrial phylogenetic results. **a** Maximum likelihood nuclear tree from the concatenated 2.41mb alignment, as in Fig. 1, but with branch lengths (RAxML). Named groups shown with same colours as in Fig. 1. Numbers show bootstrap percentages; grey branches with < 95% bootstrap support. **b** Maximum likelihood mitochondrial tree from the concatenated 12.33 kb alignment, with bootstrap percentages (RAxML). Bars show presence of clade in the ML tree for each of five subdivisions of the concatenated matrix (rRNA, followed by 4 portions of coding regions; black = clade present)

The nuclear phylogenetic results are summarized in Figs. 1 and 2a; trees are available in NEXUS format in Additional file 3. Most species relationships are resolved with strong support, and concordantly among the concatenated nuclear and species tree analyses. The independent “low quality” remnant matrices also support many of the clades. As suggested by Maddison and Hedin’s [5] results from a few genes, *Habronattus* is divided into the AAT clade (*amicus*, *agilis*, and *tranquillus* groups) and a large clade of the remaining species, within which *H. geronimoi* is sister to the rest. The *americanus* group and the VCCR clade are also resolved, and within the latter the *viridipes*, *clypeatus* and *coecatus* groups are each monophyletic.

The phylogeny has a few notable regions of uncertainty, which are also points of disagreement among the analyses. Although the previous morphological data (discussed below) provides good support for the monophyly of *Habronattus*, only one of the 1/8th nuclear partitions supports it here. The concatenated nuclear ML tree, the concatenated bootstrap consensus, three of the 1/8th partitions, and the ASTRAL tree all place *Pellenes canadensis* as sister to the AAT clade; the remnant matrices place *P. canadensis* as sister to the major *Habronattus* clade that excludes the AAT clade, while the SVDQuartets tree weakly places *P. canadensis* as sister to *H. conjunctus*. The clade including the VCCR clade plus *H. hallani*, *H. pugillis* and *H. luminosus* (“VCCR+” clade) is well supported, as is the larger clade that adds *H. cambridgei*, *H. icenoglei*, and *H. oregonensis*. However, the relationships among the latter three are unstable: the concatenated nuclear ML tree chooses (*cambridgei*, ((*icenoglei*, *oregonensis*), VCCR+)), the concatenated bootstrap consensus (*i*, ((*c*, *o*),V)), ASTRAL (*c*,(*i*,(*o*,V))), and SVDQuartets ((*c*,*i*),(*o*,V)), though the last with low bootstrap support. A few discordant placements show up in some analyses (e.g., the SVDQuartets analysis strongly places *H. tarsalis* sister to *H. americanus* and *H. sansoni*, and weakly places *H. roberti* as sister to the *viridipes* group). The unpartitioned ML analysis of the concatenated nuclear matrix yielded the same topology as in Fig. 1a, but partitioning by locus yielded the close alternative resolution (*icenoglei*, ((*oregonensis*, *cambridgei*), VCCR+). The primary concordance trees from the BCA analysis in the *americanus* group and the VCCR clade match those portions of the concatenated nuclear ML tree except for the placement of *H. virgulatus*.

### Mitochondrial phylogeny

The mitochondrial transcriptome tree (Fig. 2b; Additional file 3) is broadly concordant with the nuclear tree, concurring on the VCCR clade, the next larger clade adding *H. hallani*, *H. pugillis* and *H. luminosus*, and the next larger clade adding *H. cambridgei*, *H. oregonensis*, and *H. icenoglei*. The *americanus* group is monophyletic, as is the AAT clade. Bootstrap support values are generally high, and some key results are consistent across rRNA and four protein-coding mitochondrial subsets (Fig. 2b). The strongly supported results, however, include several notable differences with the nuclear phylogeny (Fig. 2a), including the placement of the *H. clypeatus* specimen and the relationship of the DTB clade and the *americanus* group. These will be discussed in the context of introgression.

**Fig. 3:**
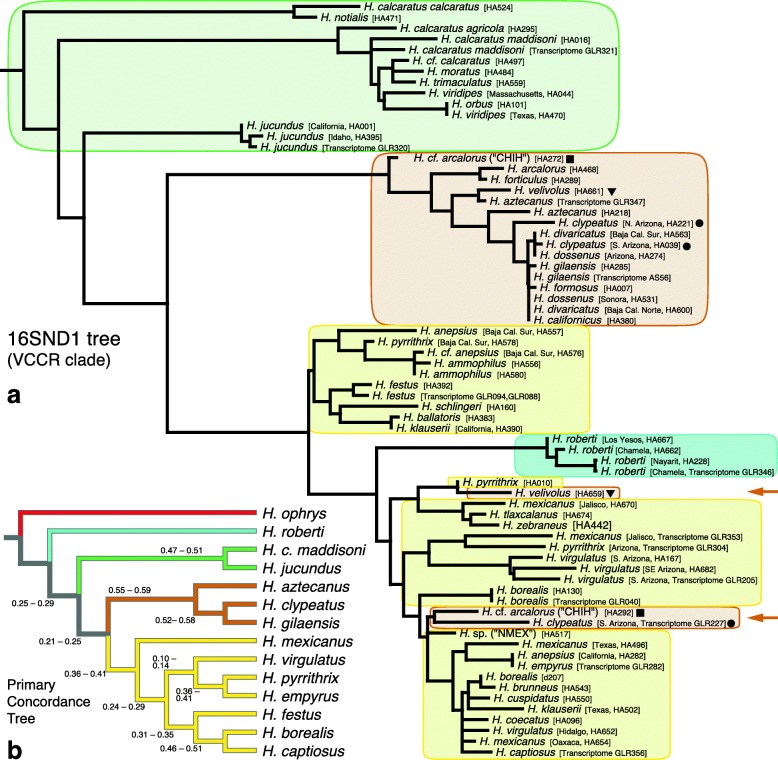
Mitochondrial and nuclear results from the VCCR clade. **a** VCCR portion of maximum likelihood tree for the 16SND1 mitochondrial region (1047 bp alignment; RAxML; branch lengths proportional to change). 160 Sanger sequenced specimens and all transcriptome specimens (marked by “Transcriptome”) are included. Tree constrained to the phylogenetic structure of the concatenated mitochondrial transcriptome phylogeny (nodes with > 90% bootstrap support only). Symbols show species polymorphic for *coecatus*-group and *clypeatus*-group type mitochondria: ● = *H. clypeatus*; ▼ = *H. velivolus*; ■ = *H.* cf. *arcalorus*. **b** BUCKy Primary Concordance tree for the VCCR clade, based on 517 genes longer than 1 kb. Node values are the CF credibility intervals for each clade. Other CFs and their credibility intervals are indicated in Fig. 5a and in Supplemental Results

The 16SND1 phylogeny (Fig. 3a; Additional file 3) of the VCCR clade, based on a combination of Sanger sequencing data and transcriptome data, generally resolves the groups but not the species. Of the eight species of the *coecatus* group represented by more than one specimen, only two appear as monophyletic on the tree (*H. ammophilus*, *H. festus*). The *clypeatus* group is not monophyletic, with three specimens (*H.* cf. *arcalorus* “CHIH*”* [HA292]; *H. velivolus* [HA659]; *H. clypeatus* [GLR227, transcriptome]) appearing within the *coecatus* group.

### Bayesian concordance analyses

**Fig. 4:**
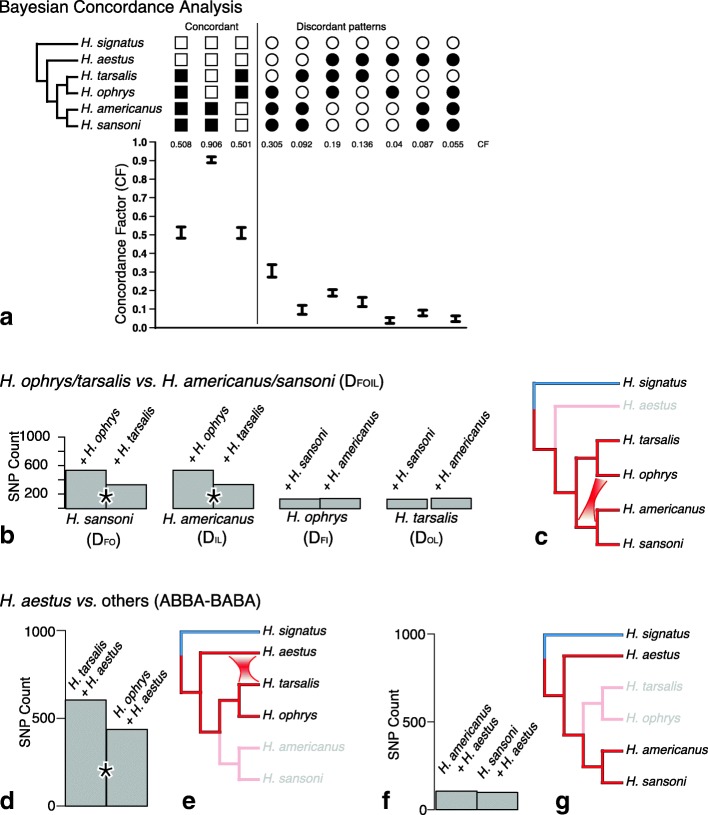
Signals of nuclear introgression in *H. americanus* species group (see Fig. 1b for summary interpretation). **a** BCA analysis using BUCKy, showing concordance factors, using Bayesian sample of trees from 679 loci. Higher CF for the discordant grouping *ophrys* + *americanus* + *sansoni* than *tarsalis* + *americanus* + *sansoni* suggests introgression. **b** Total biallelic pattern counts for all D_FOIL_ tests for introgression between *americanus* group species; * indicates significant difference at 0.05 level after Bonferroni correction. **c** Interpretation of D_FOIL_: non-neutral D_FO_ and D_IL_ values in the D-statistic signature indicate introgression between the ancestor of *H. sansoni/H. americanus* and *H. ophrys*. **d, f** ABBA vs. BABA allele patterns counts for D statistic tests for introgression in *americanus* group species; * indicates significant difference at 0.05 level after Bonferroni correction. **e, g** Interpretation: Introgression is detected between *H. aestus* and *H. tarsalis* (e); none between *H. aestus* and *H. americanus/H. sansoni* (g). Species not participating in the particular test are greyed

**Fig. 5:**
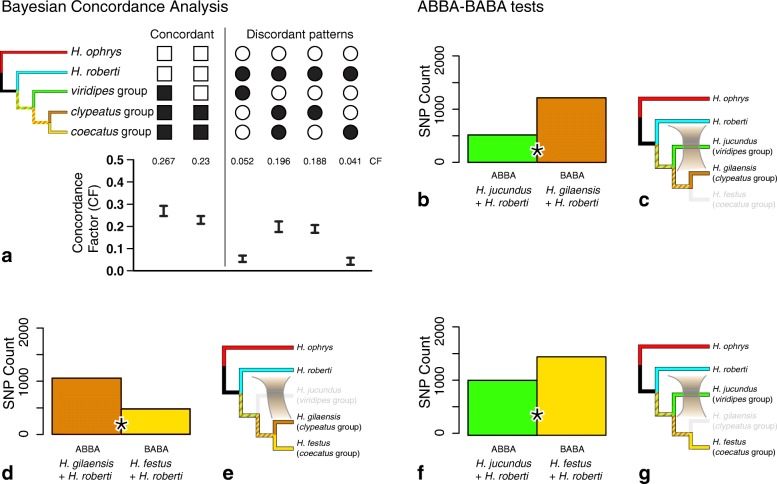
Signals of introgression between *H. roberti* and other VCCR clade members (see Fig. 1c for summary interpretation). **a** BCA analysis using BUCKy using Bayesian sample of trees from 517 loci, showing concordance factors. Higher CF for discordant grouping *roberti* + *clypeatus* group than alternatives *roberti* + *viridipes* group or *roberti* + *coecatus* group suggests introgression. **b, d, f** ABBA vs. BABA allele patterns counts for Partitioned D statistic tests for introgression between *H. roberti* and the VCCR clade. * indicates significant difference at 0.05 level after Bonferroni correction. **c, e, g** Interpretation: Introgression is detected between *H. roberti* and either the *clypeatus* group or the *coecatus* group, but the latter only when the *clypeatus* group is absent. This ghost lineage effect suggests direction introgression from the *clypeatus* group into *H. roberti*. Species not participating in the particular test are greyed

Key findings from the BCA are summarized in Fig. 4a for the *americanus* group and Fig. 5a for the VCCR clade. All additional concordance factors > 0.05 are listed in Additional file 4.

The BUCKy analysis of 679 *americanus* group loci (Fig. 4a; Additional file 4) converged with an average SD of mean sample-wide CF of 3.24 × 10^− 5^ (all 105 topologies represented among the 15 M trees sampled). The analysis supports the same *americanus* group phylogeny as the concatenated nuclear phylogeny seen in Fig. 1a. *H. ophrys* is linked to *H. americanus* and *H. sansoni* via a CF (CF = 0.305, CI = 0.272 - 0.339) that is more than twice the CF shared for the equivalent pairing *H. tarsalis* and *H. sansoni/H. americanus* (CF = 0.136, CI = 0.112 - 0.162). The BCA also found significant asymmetric support linking *H. aestus* with *H. tarsalis* (CF = 0.136, CI = 0.112 - 0.162) compared to the conflicting clade *H. aestus* and *H. ophrys* (CF = 0.39, CI = 0.023 - 0.055).

The BUCKy analysis with 517 loci for the VCCR clade (Fig. 5a; Additional file 5) converged with an average SD of mean sample-wide CF of 0.003 (4,795,750 topologies and 8177 distinct splits represented among the 15 M trees sampled). The primary concordance tree (Fig. 3b) from the VCCR-clade analysis is in agreement with the concatenated nuclear phylogeny except for the position of *H. virgulatus*, though this placement is accompanied by a very low concordance value (CF = 0.1-0.14). Two substantial conflicting (secondary) CFs place *H. roberti* with the *clypeatus/coecatus* clade (CF = 0.196, CI = 0.174 – 0.222) and with the *clypeatus* group (CF = 0.188, CI = 0.17 – 0.205). Both of these CF credibility intervals are significantly higher than those for alternative conflicting clades: *H. roberti* with the *viridipes* group (CF = 0.052, CI = 0.039 – 0.06) and *H. roberti* with the *coecatus* group (CF = 0.041, CI = 0.027 - 0.058). There are 35 very small but significant (not overlapping 0) CFs averaging 0.01 linking *H. clypeatus* with particular *coecatus* group species (see Additional file 5).

### D_FOIL_ and D-statistics tests of introgression

Asymmetries of gene-lineage sharing in the *americanus* group and the VCCR clade are seen also in the D_FOIL_ and D statistics (ABBA-BABA) results. Allele counts for these tests are listed in Additional file 6: Table S2 and Additional file 7: Table S4. Results (D values and *p* values) are reported in Additional file 7: Table S4 and Additional file 8: Table S3.

D_FOIL_ tests support introgression between *H. ophrys* and the common ancestor of *H. sansoni* and *H. americanus* (Fig. 4b, D_FO_ = 0.232 *p* < 10^− 11^, D_IL_ = 0.230 *p* = 10^− 11^, D_FI_ = − 0.036 *p* = 0.549, D_OL_ = − 0.043 *p* = 0.05). ABBA-BABA tests detect an introgression signal between *H. aestus* and *H. tarsalis*, whether the fourth species is *H. ophrys* (Fig. 4d, D= 0.160, *p* < 10^− 6^), *H. americanus* (D = 0.192, *p* < 10^− 10^), or *H. sansoni* (D = 0.165, *p* < 10^− 8^).

Introgression is detected using ABBA-BABA tests between *H. roberti* and *H. gilaensis (clypeatus* group) when the third species used for comparison i*s H. jucundus (viridipes* group) *(*Fig. 5b, D= − 0.405, *p* < 10^− 12^; interpretation in Fig. 5c*)* or *H. festus (coecatus* group) *(*Fig. 5d D = 0.377, *p* < 10^− 12^; interpretation in Fig. 5e*).* Introgression is also detected between *H. festus (coecatus* group) and *H. roberti* when *H. gilaensis* (*clypeatus)* group is excluded *(*Fig. 5f, D= − 0.177, *p* < 10^− 12^; interpretation in Fig. 5g).

**Fig. 6:**
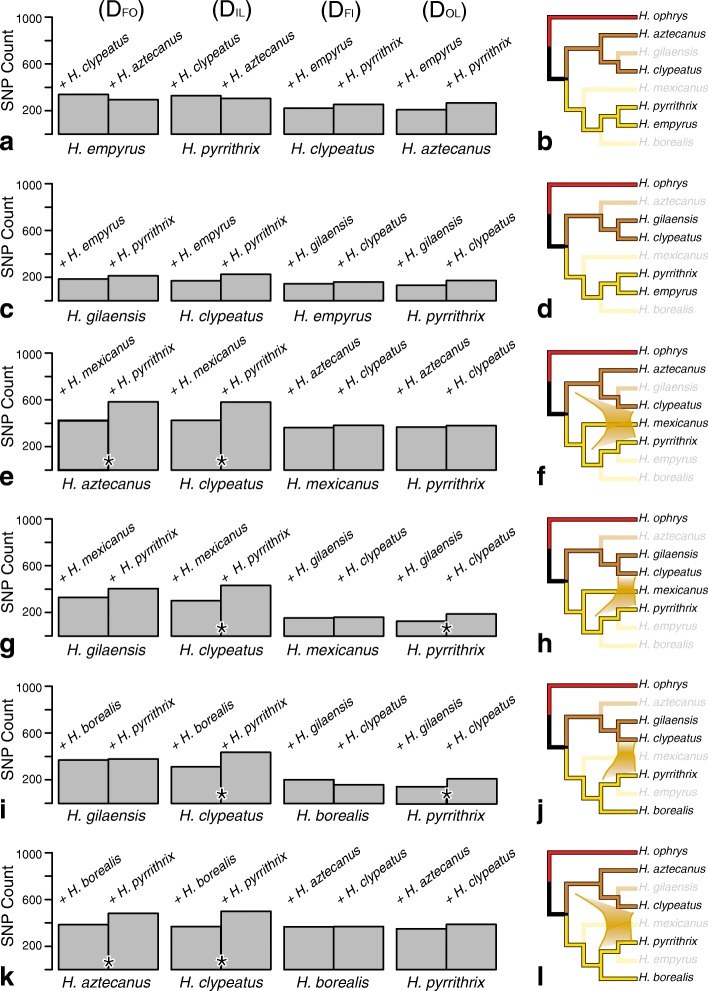
Signals of introgression between the *coecatus* and *clypeatus* groups from D_FOIL_ tests (see Fig. 1d for summary interpretation). On the left (**a, c, e, g, i, k**): total biallelic pattern counts for all D_FOIL_ tests; * indicates significant difference at the 0.05 level after Bonferroni correction. On the right (**b, d, f, h, j, l**) are phylogenetic interpretations of the tests: Introgression is detected between *H. pyrrithrix* and *H. clypeatus* (h, j) or *H. clypeatus/H. aztecanus* (f) in some but not all tests

In D_FOIL_ analyses of the *clypeatus* and *coecatus* groups (Fig. 6, Additional file 8: Table S3), introgression is detected between *H. clypeatus* and *H. pyrrithrix* when species 1 is the closely related *H. gilaensis,* and species 4 is either *H. mexicanus* (Fig. 6g, h) or *H. borealis* (Fig. 6i, j). The neutral rather than negative D_FO_ and D_FI_ values of these D_FOIL_ signatures indicate introgression with uncertain or reciprocal direction (signatures are D_FO_ = 0, D_IL_ = −, D_FI_ = 0, D_OL_ = −).

Introgression is also detected between the common ancestor of *H. aztecanus* and *H. clypeatus* and *H. pyrrithrix* when the fourth species included is the distant *coecatus* group member *H. mexicanus* (Fig. 6e, f) and the less distant *H. borealis (*Fig. 6k. l*)* because the signature shifts from D_FO_ = +, D_IL_ = -D_FI_ = 0, D_OL_ = 0 to D_FO_ = +, D_IL_ = −, D_FI_ = 0, D_OL_ = 0. With a neutral rather than positive D_FO_ value, the signature is no longer indicative of any single introgression event. However, it does hint at introgression between *H. clypeatus* and *H. pyrrithrix.* Support for introgression between *H. pyrrithrix* and the *clypeatus* group disappears when *H. empyrus* is the fourth species (Figs. 6a, c), ruling out *H. pyrrithrix*-specific introgression.

**Fig. 7:**
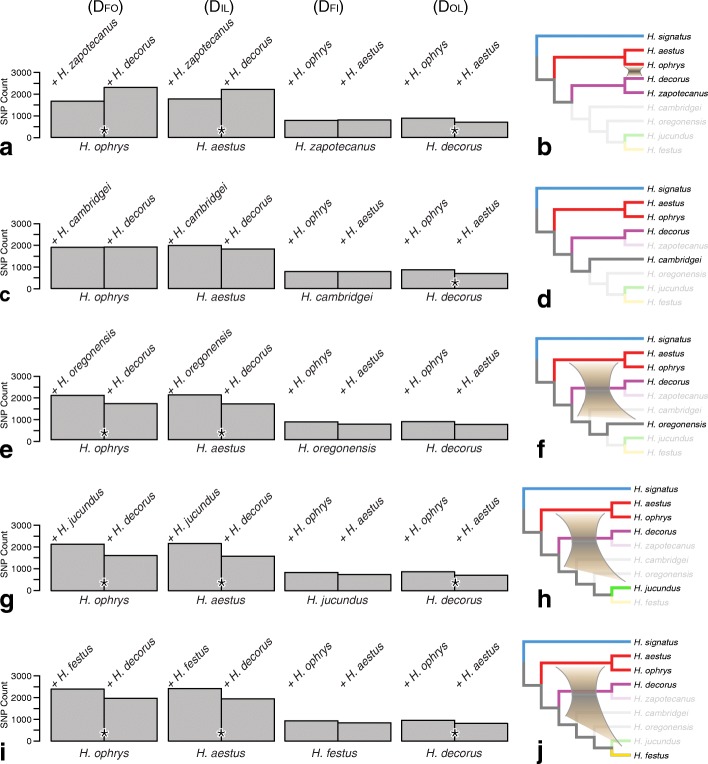
Signals of introgression deeper in the phylogeny; D_FOIL_ tests among the *americanus* group (*H. ophrys* and *H. aestus)*, *H. decorus, H. zapotecanus*, *H. cambridgei*, *H. oregonensis*, *H. jucundus*, *H. festus*. On the left (**a, c, e, g, i**) are the total biallelic pattern counts for all D_FOIL_ tests; * indicates significant difference at the 0.05 level after Bonferroni correction. On the right (**b, d, f, h, j**) are phylogenetic interpretations of the tests: no introgression was detected involving *H. cambridgei* (d), but introgression was detected between *H. ophrys* and *H. decorus* (a), and between the *americanus* group and *H. oregonensis, H. jucundus,* and *H. festus* or their ancestors (f, h, j)

D_FOIL_ and D-statistics results are presented in Fig. 7 and Additional file 6: Table S2, Additional file 7: Table S4, Additional file 8: Table S3 for the study of deeper introgression among the *americanus* group, DTB clade, and others. Several signals of introgression were detected among parts of *Habronattus* phylogeny that are now highly divergent.

## Discussion

Using transcriptome sequence data from 1877 nuclear loci, we were able to reconstruct a phylogeny for 34 species of *Habronattus* with high confidence at most nodes. Far better resolved than previous results based on just two loci [5], this robust phylogenetic framework will enable more refined interpretations of character evolution, sexual selection, and hybridization. Differences in the mitochondrial and nuclear phylogenies indicate several possible instances of mitochondrial introgression, while nuclear introgression in several regions of the phylogeny is suggested by Bayesian Concordance Analysis and D-statistics and D_FOIL_.

### *Habronattus* phylogeny

The consistency of clade support from different partitions and methods suggests that the genetic history of *Habronattus* is predominantly divergent, despite clear signs of introgression. Except for the placement of *Pellenes canadensis*, the relationships of *H. cambridgei*, *H. oregonensis* and *H. icenoglei*, and the placement of *H. virgulatus*, the phylogenetic tree shown in Fig. 1a is solidly supported as the dominant genetic history of *Habronattus* species.

We are doubtful of the placement of *P. canadensis* within *Habronattus* because of morphological support for the monophyly of *Habronattus*. Against its little-ornamented relatives *Pellenes* and *Havaika*, *Habronattus* appears well delimited by shorter first legs and a long and thin terminal apophysis (part of the male genitalia) that has a distinctive elbow on it, though the elbow is secondarily lost in the *coecatus* group [3, 5]. Despite the apparently clear synapomorphy of the elbowed terminal apophysis, the genus does not hold together as monophyletic in many of our analyses, with *Pellenes canadensis* falling inside, near *H. conjunctus* or the AAT clade. We are therefore faced with three possibilities: that the elbowed terminal apophysis arose twice, that the non-elbowed terminal apophysis of *Pellenes canadensis* represents a reversal to the ancestral state, or that *Pellenes canadensis* and other members of its subgenus *Pellenattus* form a close sister group to *Habronattus*, with extremely short branches separating their early divergence, leading to difficulties in resolving the deeper relationships, especially given that the outgroup *Evarcha* is phylogenetically distant, in a separate subtribe (the Plexippina [37]). The choice may be resolved by having better sampling of outgroups among the Harmochirina. Because *Pellenes* species are also distributed in Asia [61] and Europe [62], a broader global sample of *Pellenes* specimens and other closely related groups (e.g., *Harmochirus*) should be included to better tease apart relationships at the base of the *Habronattus* tree and determine if the genus is monophyletic. We expect, however, that addition of outgroups would not change the well-supported relationships within the major clade from *H. paratus* and *geronimoi* to the VCCR clade. The mtDNA tree (Fig. 3a), which included the additional outgroups *Havaika*, *Harmochirus*, *Bianor*, and others, is largely consistent with Fig. 1.

The reconstructed phylogeny is generally consistent with morphology [3] and the previous results from two genes [5]. The major clade whose males have fringed first legs and modified third legs (the VCCR clade) is intact as monophyletic, as are the three contained groups first recognized by morphology (*viridipes*, *clypeatus*, and *coecatus* groups). In this regard, the phylogeny is more concordant with morphology than that of Maddison and Hedin [5], whose analyses gave unexpected placements for *H. jucundus* (*viridipes* group) and split the VCCR clade. The *americanus* group, with distinctive genitalia and relatively long first legs, is monophyletic in the transcriptome phylogeny. The *agilis*, *amicus* and *tranquillus* groups hold together as the AAT clade, distinguished by compact bodies, a relatively far-rotated bulb of the male palp, and a tendency for dwelling above the ground in shrubs. As expected from the results of [5] the AAT clade is sister to the remainder of the genus, within which *H. paratus* (in the mitochondrial tree) is most basal with respect to the bulk of species, and *H. geronimoi* next.

The nuclear phylogeny provides new resolution of mid-level relationships in *Habronattus*. The strong support for the clade of *H. decorus*, *H. altanus*, *H. zapotecanus* and *H. chamela* indicates that their species groups (*decorus*, *texanus*, and *banksi* groups) form a clade, here called the DTB clade. The previously intractable *H. hallani* is strongly supported as sister to *H. luminosus* and *H. pugillis.* The placement of these three species as sister to the VCCR clade is novel, as is their collective relationship with *H. icenoglei*, *H. oregonensis*, and *H. cambridgei*. For the first time there are well-supported clades within the *coecatus* group: *H. festus*, *H. captiosus* and *H. borealis* together (100% bootstrap support) will be referred to as the Northern clade (all specimens in this clade were collected in Canada), while *H. empyrus* and *H. pyrrithrix* are sisters (100% bootstrap support), forming the Southern clade (they are both found in the southern USA neighboring Mexico). *H. virgulatus* is sister to the Northern *coecatus* clade with high support (97% bootstrap support). The relationships among the subgroups of the VCCR clade are resolved with good bootstrap support: the *viridipes*, *clypeatus*, and *coecatus* groups form a clade (nuclear bootstrap = 83%), with *H. roberti* as sister to them; the *clypeatus* and *coecatus* group are sisters (100%); and each of the *viridipes*, *clypeatus* and *coecatus* groups is monophyletic. *H. jucundus* groups with *H. calcaratus* rather than with the *oregonensis* group (a poorly supported relationship found in [5]), confirming that the *viridipes* group is in fact monophyletic. The internal relationships of the *americanus* group also have high bootstrap support.

Most of these highly supported relationships are also replicated in different nuclear subsets (see subset support summaries at the nodes in Fig. 1a), suggesting that the phylogenetic signal for most branches is robust even with less data. However, there are a few nodes with high bootstrap support that are unstable across nuclear subsets. *H. roberti*, while well supported overall as sister to the rest of the VCCR clade, also groups with the *clypeatus* group in some nuclear subsets. *H. virgulatus* departs from its dominant concatenated nuclear position (bootstrap support = 97%) to group with either the Southern *coecatus* clade or as a basal branch of the *coecatus* group in some nuclear subsets. *H. ophrys* is positioned as the sister to *H. sansoni* and *H. americanus* in half of all data subsets, despite being sister to *H. tarsalis* (bootstrap support = 100%) in the concatenated nuclear phylogeny.

Even if *Habronattus* has a predominantly divergent history with a clear modal gene tree, there could still be a broad scatter of discordant gene trees through incomplete lineage sorting or hybridization. Such discordance is expected from the group’s youth (perhaps less than 5 million years, [17]), and is seen in our data. For instance, the VCCR clade has very low dominant CFs from the Bayesian Concordance Analysis (Fig. 3b), potentially indicating that the group is still in the early stages of divergence with widespread incomplete lineage sorting and possibly also ongoing hybridization. The indications of incomplete divergence are strongest in the *coecatus* group, where there are 196 secondary (conflicting) CFs that are significant (greater than 0), though most are very small and not clearly indicating ILS vs. introgression. The *americanus* group, on the other hand, has stronger genetic concordance (high dominant CF values) even though it is equally recently diverged ([12], fig. 4a). However, such differences in concordance could reflect differences between the groups in the density and locations of sampling, and not necessarily a difference in their evolutionary dynamics.

### Introgression in *Habronattus*

Maddison & Hedin [5]’s evidence for introgression was the presence in two *clypeatus* group specimens of mitochondrial 16SND1 more closely related to that of the *coecatus* group, close enough to argue against ILS as the source of discord. Our data from full mitochondrial transcriptomes indicate another case of a *clypeatus*-group specimen with mitochondria falling with the *coecatus* group, and hint of possible introgression elsewhere in the phylogeny (in the *americanus* group, in the VCCR clade, and deeper in the tree).

Our tests using BCA, D_FOIL_, and D-statistics indicate that introgression extends to the nuclear genome as well. We detected nuclear introgression among closely related species, as also found by [21], ([4, 11]; *pugillis* group), and ([63]: *H. americanus*). However, our analyses also find clear signals of more distant nuclear introgression, among species groups: between *H. roberti* and the *clypeatus* group, between the *clypeatus* and *coecatus* groups, and apparently among even more distantly related species. We will consider the evidence for introgression separately for different regions of the phylogeny.

#### Introgression within the *americanus* group

The nuclear phylogeny, by grouping *H. americanus* with *H. sansoni*, and *H. tarsalis* with *H. ophrys*, is in accord with the informally-recognized distinction of the *americanus* group into the *americanus* subgroup and the *tarsalis* subgroup. The former typically have black first legs without lateral fringes and are usually found on ground covered in rocks, sticks and litter (*H. americanus*, *H. sansoni*, *H. waughi* (Emerton, 1926), *H. bulbipes* (Chamberlin & Ivie, 1941), *H. kubai* (Griswold, 1979)); the latter have yellow, green or brown first legs with lateral fringes, and are typically found on grassy ground (*H. tarsalis*, *H. kawini* (Griswold, 1979), *H. mustaciata* (Chamberlin & Ivie, 1941), *H. ophrys*, *H. gigas* Griswold, 1987). The mitochondrial phylogeny (Fig. 2b), however, strongly supports a sister group relationship between *H. ophrys* and *H. americanus* to the exclusion of *H. sansoni*. On its own, this result could be explained by either introgression or incomplete lineage sorting. However, two introgression events in the *americanus* group were detected in the nuclear data*.*

Introgression among *H. ophrys*, *H. americanus* and *H. sansoni* is indicated by both BCA and D_FOIL_ (Fig. 4), linking *H. ophrys* to *H. americanus* and *H. sansoni* collectively, with no sign of a preferred link to either *H. americanus* or *H. sansoni*. However, *H. ophrys* and *H. americanus* in particular are resolved as sister taxa by several nuclear data subsets (Fig. 1a) and in the mitochondrial phylogeny (Fig. 2b). Such a pattern could arise by multiple introgression events involving *H. ophrys* with either the ancestor of *H. americanus* and *H. sansoni*, or one of the two, or both separately, possibly combined with introgression between *H. americanus* and *H. sansoni*. *H. ophrys* is currently sympatric with *H. americanus* along the Pacific Coast of British Columbia, Washington and Oregon, and so may have had more opportunity to hybridize with it than with the allopatric *H. sansoni*. Introgression involving *H. ophrys* could explain the distribution of one morphological trait that is discordant with the nuclear phylogeny: tufts above the front pair of eyes, male ornaments unique in *Habronattus* to the *americanus* group, are found only in *H. sansoni* and close relatives (*H. kubai,* some *H. americanus* populations), and in *H. ophrys.*

Also found in both the BCA and D statistic tests are signals of introgression involving *H. aestus* and *H. tarsalis* (Fig. 4f). The BCA found substantial, asymmetric support linking *H. aestus* with *H. tarsalis* only compared to the conflicting clade *H. aestus* and *H. ophrys* (Fig. 4d). This pattern is also supported by ABBA-BABA tests, which detected an introgression signal between *H. aestus* and *H. tarsalis* (Fig. 4e).

Results from the BCA provide some insights into the extent of nuclear introgression. The genome-wide concordance factor of a clade is an estimate of the proportion of the genome for which the clade is true [34]. The primary CF and species tree put *H. ophrys* and *H. tarsalis* together, but the CF for the discordant *H. ophrys*/*H. americanus*/*H. sansoni* clade is 0.305, indicating about 30% of the genome has those conflicting relationships. Of this 30%, 9% can be explained as due to ILS, given that the alternative *H. tarsalis*/*H. americanus*/*H. sansoni* clade has a CF of 0.092. This leaves a difference of 21% that cannot be explained by ILS, suggesting that as much as 21% of the genome could have been introgressed between *H. ophrys* and *H. americanus*/*H. sansoni*. A similar argument suggests up to 10% introgression between *H. aestus* and *H. tarsalis.*

#### Introgression between *H. roberti* and other VCCR groups

Ambiguity in the placement of *H. roberti* in the phylogenetic analyses hints to possible discordance. The concatenated nuclear tree and ASTRAL place *H. roberti* sister to the remainder of the VCCR clade (Fig. 1), but some partitions and SVDQuartets give other relationships, e.g. as sister to the *clypeatus* group, sister to the *clypeatus* plus *coecatus* groups, sister to the *viridipes* group, or sister to the *coecatus* group. The primary concordance tree from BCA places *H. roberti* as sister to the remainder of the VCCR clade, but with a weak concordance factor (CF = 0.267; Fig. 5a).

Both BCA and D statistics suggest that the ambiguity in the placement of *H. roberti* is not due solely to short branches or incomplete lineage sorting, but involves a strong component of discordance from introgression. Discordant patterns show a strong asymmetry toward *H. roberti* plus the *clypeatus* group (BCA, Fig. 5a; ABBA-BABA, Figs. 5b-g) rather than the alternatives of *H. roberti* plus the *viridipes* group or the *coecatus* group. When the *clypeatus* group is excluded from tests, a signal for introgression is seen between *H. roberti* and the *coecatus* group (Figs. 5f and g), but this is likely as ghost lineage effect ([32, 63]), as this signal shifts to the *clypeatus* group when included (Figs. 5d and e). This ghost lineage effect suggests a direction of the introgression: from the *clypeatus* group into *H. roberti* [59]. By Figs. 5d and e, the strong signal is between *H. roberti* and the *clypeatus* group. If *H. roberti* were the donor species instead, into the *clypeatus* group, then we would expect to see sharing of alleles between just those two, failing to predict the ghost effect of sharing between *H. roberti* and the *coecatus* group (Figs. 5f and g). Following a similar argument to that given for the *americanus* group, the excess CF difference of 0.14 for the discordant clade *H. roberti* plus the *clypeatus* group compared to contrasting discordant patterns (CF 0.188 compared to 0.04-0.05) could suggest that 14% of the genome of *H. roberti* is introgressed from the *clypeatus* group.

The muddled nature of genetic relationships of *H. roberti* is echoed in its phenotype. The species was considered part of the *viridipes* group by Maddison and Hedin [5] based on its sharing the latter’s synapomorphy of a raised ridge of setae between the male’s posterior eyes [35]. However, *H. roberti* has a clearly visible checkered pattern of pigment in the male anterior median eyes, otherwise known only from the *clypeatus* group [35]. It also has a dark medial ventral abdominal stripe, as in the *clypeatus* group. Males from some populations of *H. roberti* have red-purple bumps on their third leg’s patella [35], a trait found otherwise only in *clypeatus* group species *H.* cf. *arcalorus* “CHIH”, *H. formosus* (Banks, 1906)*,* and *H. velivolus* Griswold, 1987 (a species sympatric with *H. roberti*). If *H. roberti* is sister to the VCCR clade as per the nuclear phylogeny, those traits in which it resembles the *viridipes* group could be ancestral to the VCCR clade, with its *clypeatus*-group traits acquired by introgression.

#### Introgression between the *coecatus* and *clypeatus* group

Our finding of a third case of mitochondrial sequences in the *clypeatus* group falling in the *coecatus* group (Fig. 3a) adds to the previous results of Maddison and Hedin [5]. Our *H. clypeatus* specimen has a mitochondrial genome nestled well within the *coecatus* group, strongly supported by the whole mitochondrial transcriptome (Fig. 2b). Maddison and Hedin [5] found specimens of *H. velivolus* and *H.* cf. *arcalorus* “CHIH” with 16SND1 sequences closely resembling those of the *coecatus* group. In each case, these species are polymorphic, with specimens from the same or other locations showing typical *clypeatus*-group mitochondrial genes. ILS is formally a possible explanation, requiring that the mitochondrial lineage dominant in the *clypeatus* group went extinct or unsampled in the *coecatus* group. However, if ILS were the cause of discord, the mitochondrial lineages shared between unusual *clypeatus* group specimens and the *coecatus* group would have to extend deeper than the common ancestor of the 35 described species in the clade containing the *clypeatus* and *coecatus* groups. However, the sequence divergences are small — for example, the divergence between *H. velivolus* HA659 (*clypeatus* group) and *H. pyrrithrix* HA010 (*coecatus* group) is about the same as that among the three *H. virgulatus* specimens from Arizona. If divergence between HA659 and HA010 mitochondria was prior to the split of the two species groups, this would suggest that either the three *H. virgulatus* mitochondria also extend that deep and just by luck managed to sort themselves into three specimens of the same morphospecies, or the rate of sequence divergence in *H. virgulatus* has drastically sped up. Recent mitochondrial introgression is a simpler explanation.

A signal for nuclear introgression between the *coecatus* and *clypeatus* groups, possibly many events, is found via D_FOIL_ tests. Introgression is detected between *H. pyrrithrix* and *H. clypeatus* (Figs. 6g–j), and between the common ancestor of *H. aztecanus* and *H. clypeatus* and *H. pyrrithrix (*Figs. 6e and f*)*. However, these signals are lost whenever *H. empyrus,* the sister taxon of *H. pyrrithrix,* is included in the test, ruling out *H. pyrrithrix*-specific introgression. The signal is also partially lost, producing D_FOIL_ signatures inconsistent with a single introgression event, when *H. mexicanus (*Fig. 6e) is replaced by *H. borealis* (Fig. 6 and k) as the *coecatus* species used for comparison. The complex pattern of D_FOIL_ signatures that vary depending on the species included for comparison could be explained by multiple introgression events. It does not indicate a clear direction (*coecatus* group into *clypeatus* group or vice versa). However, the lack of an introgression signal in Figs. 6a–d helps pinpoint the origin of the introgression signal in the *coecatus* group to an unidentified ghost taxon from the Southern clade that is closely related to *H. pyrrithrix*.

Despite the clear signal of introgression from D_FOIL_, the BCA does not show any concordance factors of greater than 0.05 intermixing the *clypeatus* and *coecatus* groups. Although there are 35 significant (not overlapping 0) CFs that link *H. clypeatus* with *coecatus* group species, these are so small (averaging 0.01) that they cannot be attributed to introgression — low CF values are likely to be overestimated [54], and are just as likely to be a result of ILS or noisy gene trees [33].

The mitochondrial and nuclear results could be an indication of frequent introgression between the *clypeatus* group (containing 10 species) and *coecatus* group (24 species). This is unexpected, as these clades differ substantially and consistently in morphology and courtship behaviour [5, 6, 64]. The male courtship display is highly complex in both groups, but differs in morphological ornaments, behaviours, and acoustic signals [6, 64]. Genital morphology is also quite different in males and females of the two groups [3]. Species of the *coecatus* group lack an elbow on the male palp’s terminal apophysis, present in all *clypeatus* group species [3]. The *clypeatus* group has a male tibial apophysis that is thinner and hook-like; the *coecatus* group thicker and more triangular. The possibility of hybridization despite these substantial differences related to mating is worth noting. Because of a predicted evolutionary lag between male sexually selected traits and female preferences [65], species-specific differences in male mating traits do not always result in reproductive isolation [66]. However, given the considerable phylogenetic distance and differences between *clypeatus* and *coecatus* groups, isolation would have been expected.

#### Introgression signal as artifact?

Our inference of nuclear introgression is based on a dataset sorted into proposed orthologs and assumed to be free of contamination. Were some of our “loci” a blend of different paralogs, some resulting gene trees may appear (falsely) to reveal introgression. However, these “loci” discordant with the primary species tree would not be expected to have a consistent direction of discord, instead adding non-directional noise, unless we suppose an elaborate scenario (e.g. chromosome duplication in a shared ancestor, with the alternatives marked and later deleted wholesale in different descendants). There is no cytogenetic reason to suspect large-scale duplications: except for *H. banksi* (Peckham & Peckham, 1901) and *H. zapotecanus*, all *Habronattus* have the same number of chromosome arms and approximately the same size of chromosomes (71 species studied in Maddison & Leduc-Robert [12], including most reported here). While paralogs might have survived our filters, it is unlikely that they generated our signals of introgression.

A more serious concern would be cross-sample contamination [67], which could mimic introgression closely. Ballenghien et al. [67] noted that contamination was most likely between samples sharing a batch at a sequencing facility. Our samples were run in five batches separated by many months or in different facilities: (1) *H. festus* and *ophrys*; (2) *H. aztecanus*, *cambridgei*, *captiosus*, *chamela*, *hirsutus*, *mexicanus*, *paratus*, *roberti*, and *zapotecanus*; (3) *H. signatus* and *ustulatus*; (4) *H. gilaensis*; and (5) all other taxa. Of our four primary conclusions of introgression (Figs. 1 b, c and d), one can be easily defended: *H. ophrys* was in a different sequencing batch from *H. americanus* and *H. sansoni*. The other tests of introgression could have been compromised by within-batch contamination. For example, *H. roberti* shared a sequencing batch with *H. aztecanus*, a *clypeatus* group member sympatric with *H. roberti*, and thus a possible source of the patterns of Fig. 5 through either contamination or introgression. However, although contamination should be acknowledged as a possibility, it is unlikely to be the source of introgression signals in *H. roberti*. Most critically, contamination from *H. aztecanus* would predict a pattern of biased discord similar in nuclear and mitochondrial genomes, while introgression would predict a different pattern in the mtDNA, with a single clear tree and no biased discord among sites. The latter is what we see. The nuclear ABBA-BABA test of Figs. 5d for *H. festus* - *gilaensis* - *roberti* - *ophrys* shows a strong bias of 1051 for ABBA versus 476 for BABA (Additional file 7:Table S4), but the corresponding test on mtDNA (reordered *H. festus* - *roberti* - *gilaensis* - *ophrys* to match the inferred mitochondrial tree) shows no bias, with 89 sites ABBA and 88 BABA (and 215 BBAA). There is no sign of a minority signal in the mtDNA, against the predictions of contamination. In addition, our protocol would have scored as ambiguous, and thus excluded from D statistics tests, any nucleotide unless it reached more than 70% prevalence among reads at a site in our protocol. Ballenghein et al.’s [67] data suggest it rare for a contaminant to achieve such high prevalence: just 6 of their 446 *cox1* samples exceed a 7:3 ratio of unexpected:expected reads (their fig. 2b). A frequency of 6/446 is not high to yield the biases we observed (Additional file 7: Table S4). Finally, *H. roberti* is not an outlier in percentage of sites with ambiguous calls. Similar arguments can be given for the other conclusions of introgression in Fig. 1, and thus contamination is not a likely alternative explanation.

#### Signals of broader introgression

Ancient introgression deeper in *Habronattus* phylogeny is suggested by both the mitochondrial phylogeny and D statistics. Mitochondrial introgression between the *americanus* group and the DTB clade could explain why these groups form a clade in the mitochondrial transcriptome phylogeny (Fig. 2b) but are separate and distant clades in the nuclear phylogeny (Fig. 2a). When we explored this further in the nuclear data with D-statistics, we found introgression signals shared not only between the *americanus* group and *H. decorus*, but also with many more distant species (Fig. 7). These results are difficult to interpret because the phylogenetic distance obscures the donor and recipient lineages, and multiple introgression events could create conflicting signals.

#### Potential drivers of hybridization

Our results indicate a clear genomic signature of introgression in *Habronattus* among species groups with strong phenotypic differences in courtship and genitalia, differences which might have been expected to provide reproductive isolation. We found introgression not only between the *clypeatus* and *coecatus* groups (as in [5]), but also between *H. roberti* and the *clypeatus* group, and possibly other more distant species.

Evidence for directional introgression involving the *coecatus* group may hint at what processes have driven hybridization despite divergent morphology and behaviour. Courtship of the *coecatus* group is strikingly complex [6], more complex than any other known in *Habronattus*. Mitochondrial data suggest introgression from the *coecatus* group into the *clypeatus* group (Figs. 2b, 3a), but nuclear data show hints of multiple introgressions with no clear directionality (Fig. 6). Directionality of mitochondrial introgression but not nuclear introgression could be the result of sex-biased hybridization, driven by a difference in female discrimination [68] causing *coecatus* females to sometimes hybridize with *clypeatus* group males but not the other way around. In some *Habronattus* (outside of the *coecatus* and *clypeatus* groups), females have been found to prefer foreign males from divergent populations (*H. pugillis*; [7]). Such xenophilia could be a consequence of an arms race between the sexes [27] involving sensory exploitation: males would evolve novel exploitative signals; females evolve resistance to their own males, but not to new signals evolved in foreign lineages to which the females have not been exposed, but which share common sensory biases [6, 7]. If females of a species group (e.g. the *coecatus* group) are particularly susceptible to novel signals, then this could lead both to higher courtship complexity in that group, and to their females’ tendency to donate mitochondria to distant species. Thus, the tendency to hybridize might correlate positively with more complex courtship behaviour. However, other factors could promote asymmetric introgression. Hedin and Lowder [21] suggested asymmetrical overlap in body size distributions could explain the directional introgression they found in the *amicus* group of *Habronattus.* Demographic characteristics of hybridizing species [23], such as differences in population size [69] and dispersal behaviours [70], could also cause asymmetric introgression.

#### Evolutionary consequences of introgression

Introgression in *Habronattus* may have been frequent enough to result in detectable nuclear admixture, but infrequent enough so as not to be a homogenizing force reducing genetic and morphological diversity [24]. For instance, Blackburn and Maddison [71] found evidence for gene flow among parapatric subpopulations of *H. americanus*, and yet they showed consistent courtship display differences. Both substantial introgression and rapid diversification are found in the species-rich *americanus* group (12 species) and the VCCR clade (42 species), both of which have remarkable diversities of male ornaments. In the VCCR group, as much as 14% of derived alleles are shared between distantly related *clypeatus*/*coecatus* groups and *H. roberti* due to introgression, while closely related *americanus* group species *H. ophrys* and *H. americanus/H. sansoni* share up to 21% of derived alleles as a result of introgression.

It is unclear whether introgression has played a creative evolutionary role in *Habronattus*, either by promoting diversification or by influencing the phylogenetic distribution of traits. Distant introgression, which we detected in several clades, is more likely to have adaptive effects on lineages because novel and potentially adaptive genetic combinations are more likely to form as a result of introgression when there is more time to accumulate genetic differences [72, 73]. Introgression could also create adaptive potential by increasing the standing variation of hybridizing lineages, which could facilitate subsequent diversification [73]. There are an increasing number of documented cases of adaptive introgression [74–76] and introgression-facilitated diversification in animals [77, 78]. Given the strength of sexual selection in *Habronattus* [1, 2, 11], loci implicated in sexually selected traits could be under strong selection if they were exchanged between hybridizing species. We have suggested two courtship traits in *Habronattus* whose phylogenetic distributions could be explained by introgression: the red-purple bumps on the third legs of males of *H. roberti* and members of the *clypeatus* group, and the distinctive “eyebrows” of *H. ophrys* and *H. sansoni*. A denser phylogenetic sample of *Habronattus* species will be needed to better resolve whether such similarities discordant with the species tree are best explained by distant introgression.

## Conclusions

We produced a highly resolved phylogeny of *Habronattus* and determined the contributions of hybridization and incomplete lineage sorting to genetic discordance in the group*.* We found that hybridization has been common in *Habronattus* phylogeny*,* and has resulted in considerable nuclear introgression in some instances (e.g., the *americanus* group, *H. roberti*) and lesser nuclear introgression accompanied by strong mitochondrial introgression in others (i.e., among the *coecatus* and *clypeatus* groups). However, we were unable to detect specific lineages and direction of introgression in many cases, for which a denser sample of species will be important. Widespread introgression between both distant and closely related species indicates that only partial reproductive isolation has evolved across much of the *Habronattus* phylogeny. Introgression has occurred between *Habronattus* species groups with divergent male ornaments and courtship behaviours. Further research should focus on determining the extent of introgression’s contribution to adaptation and diversification. In particular, widespread introgression in the highly diverse VCCR clade could be indicative of a correlation between introgression and diversification.

## Additional files

Additional file 1: **Table S1.** Summary of RNA-sequencing and transcriptome assemblies for all species (except DNA sequencing of *H. paratus*). (PDF 616 kb)

Additional file 2: NEXUS files of aligned matrices (five text files, NEXUS format, totaling 131 mb, compressed to a single zip file of 12 mb) (ZIP 15390 kb)

Additional file 3: NEXUS file of the phylogenetic trees from various analyses (text, NEXUS format 250 kb) (TXT 244 kb)

Additional file 4: Concordance file for *americanus* group. (TXT 74 kb)

Additional file 5: Concordance file for VCCR clade (text 4.3 mb) (TXT 4182 kb)

Additional file 6: **Table S2.** Counts of alleles shared among *Habronattus* species used for D_FOIL_ tests. (PDF 405 kb)

Additional file 7: **Table S4.**. Patterson’s D statistic results and counts of allele patterns. (PDF 455 kb)

Additional file 8: **Table S3.** D_FOIL_ results. (PDF 476 kb)

## Abbreviations

AAT: The clade of species including the *agilis*, *amicus*, *tranquillus* groups.
BCA: Bayesian Concordance Analysis.
bp: Base pair (1 nucleotide).
CF: Concordance factor.
CI: Credibility interval.
DTB: The clade of species including the *decorus*, *texanus*, and *banksi* groups.
ILS: Incomplete lineage sorting.
kb: Kilobase (1 thousand nucleotides).
Mb: Megabase (1 million nucleotides).
ML: Maximum likelihood.
MS: “Missing Species”, a subset of the data.
NL: “Noncoding Loci”, a subset of the data.
VCCR: the clade of species including the *viridipes*, *coecatus*, *clypeatus*, and *roberti* groups.
