# Supplementary material for: Phylogeny with introgression in Habronattus jumping spiders (Araneae: Salticidae)
Source: BMC Evol Biol. 2018 Feb 22;18:24. doi: 10.1186/s12862-018-1137-x (PMC5824460; doi:10.1186/s12862-018-1137-x)
Supplement: Supplementary file 1 — Table S1. Summary of RNA-sequencing and transcriptome assemblies for all species (except DNA sequencing of H. paratus). (PDF 616 kb) [file 12862_2018_1137_MOESM1_ESM.pdf]

Table S1. Summary of RNA-sequencing and transcriptome assemblies for all species (except DNA sequencing of *H. paratus*): NCBI Sequence Read Archive accession numbers, total number of sequenced reads after filtering and trimming, percentage of reads mapped to nuclear and mitochondrial references, average sequencing depth for all de novo and reference-based transcriptome assemblies, and number of contigs.

Species	SRA accession	# reads	%reads to nuc.	%reads to mtDNA	% GC	Avg. nuclear depth	Avg. mtDNA depth	Total contigs
H. aestus	SRR6381079	9,099,515	66.1	14.0	35	63	16,711	12,460
H. altanus	SRR6381065	3,773,233	61.0	13.7	36	45	6,632	7,310
H. americanus	SRR6381060	5,053,616	70.3	11.9	37	53	7,721	9,004
H. aztecanus	SRR6381090	3,111,757	92.5	0.2	35	49	7,293	5,767
H. borealis	SRR6381063	14,702,204	64.0	16.6	38	89	31,830	13,992
H. c. maddisoni	SRR6381069	4,345,865	56.0	25.3	35	54	14,496	6,121
H. cambridgei	SRR6381082	6,216,145	64.9	16.9	36	65	14,220	8,799
H. captiosus	SRR6381085	5,013,439	62.8	16.8	35	46	11,492	9,701
H. chamela	SRR6381083	4,448,302	70.8	13.8	36	48	8,382	8,290
H. clypeatus	SRR6381056	9,419,193	64.8	15.4	37	78	19,106	10,554
H. conjunctus	SRR6381064	12,726,652	63.7	15.3	35	94	25,324	10,354
H. decorus	SRR6381070	5,637,415	56.7	20.3	34	48	15,069	8,747
H. empyrus	SRR6381086	4,639,425	59.0	19.6	35	57	11,904	11,666
H. festus	SRR6381057,84	61,707,379	67.0	15.9	33	174	132,740	28,846
H. geronimoi	SRR6381071	7,742,035	67.8	13.7	36	79	13,571	8,833
H. gilaensis	SRR6381089	7,976,865	70.0	8.7	37	60	13,666	15,564
H. hallani	SRR6381072	6,789,244	63.2	16.5	36	57	14,366	9,769
H. hirsutus	SRR6381077	3,722,052	58.1	14.9	36	42	7,517	6,238
H. icenoglei	SRR6381074	6,909,819	71.7	9.3	37	90	7,720	7,193
H. jucundus	SRR6381068	6,007,999	65.1	16.0	37	50	12,504	9,176
H. luminosus	SRR6381087	10,564,035	71.2	8.9	38	89	12,169	10,490
H. mexicanus	SRR6381088	5,159,443	59.5	21.1	36	50	14,828	8,192
H. ophrys	SRR6381058,59	61,994,076	73.1	15.9	33	111	86,313	51,143
H. oregonensis	SRR6381078	5,299,397	62.9	16.0	37	54	11,821	8,700
H. paratus	SRR6381080	11,963,220	31.6	0.0004	30	64	60	9,109
H. pugillis	SRR6381073	6,812,739	68.4	16.0	37	74	13,985	8,494
H. pyrrithrix	SRR6381067	11,977,280	65.9	15.4	36	93	23,939	10,828
H. roberti	SRR6381066	2,654,337	63.1	17.1	35	43	6,188	3,746
H. sansoni	SRR6381055	4,691,985	65.2	15.1	33	44	9,010	9,011
H. signatus	SRX652504	63,057,403	64.8	10.0	37	87	48,192	18,336
H. tarsalis	SRR6381061	4,748,864	63.6	11.5	37	52	7,095	7,671
H. ustulatus	SRX763246	60,262,898	69.5	7.0	37	82	28,862	18,336
H. virgulatus	SRR6381062	5,483,627	60.1	18.8	33	60	13,256	7,671
H. zapotecanus	SRR6381081	11,275,197	50.0	10.3	40	65	15,759	11,939
E. proszynskii	SRR6381076	10,771,679	38.5	21.5	37	60	30,009	5,416
P. canadensis	SRR6381075	9,145,521	55.7	15.8	36	65	18,840	9,426
