# Supplementary material for: Phylogeny with introgression in Habronattus jumping spiders (Araneae: Salticidae)
Source: BMC Evol Biol. 2018 Feb 22;18:24. doi: 10.1186/s12862-018-1137-x (PMC5824460; doi:10.1186/s12862-018-1137-x)
Supplement: Supplementary file 2 — NEXUS files of aligned matrices (five text files, NEXUS format, totaling 131 mb, compressed to a single zip file of 12 mb) (ZIP 15390 kb) [file 12862_2018_1137_MOESM2_ESM.zip]

Table S2. Counts of alleles shared among *Habronattus* species used for D_{FOIL} tests. For species identities see row with corresponding Figure in Supplementary Table 3.

Fig.	AAAAA	BBBBA	BBAAA	AABBA	ABABA	ABBAA	ABBBA	BAABA	BABAA	BABBA	BBABA	BBBAA	BAAAA	ABAAA	AAABA	AABAA
***americanus* group**																
4b	1144994	33651	1257	678	34	49	74	21	37	63	266	468	375	562	1700	1325
***clypeatus* and *coecatus* groups**																
6a	1305813	17312	2452	2108	55	55	248	37	52	188	300	346	2368	1846	3122	2023
6c	1505981	17856	2576	2813	42	33	160	21	30	124	231	176	1192	1235	1711	2373
6e	1261335	16612	1950	1550	105	91	300	120	102	279	569	358	3017	2014	2951	4534
6g	1399985	16507	2264	1709	68	38	145	34	40	99	471	333	1072	1140	2806	4400
6i	1598152	18920	2600	2072	80	39	144	31	65	127	439	350	1208	1324	3004	3711
6k	1344100	17806	2068	1787	89	78	340	75	88	312	490	338	3218	2121	2860	3650
**Deeper introgression**																
7a	1433854	31209	6391	2939	189	225	347	231	167	431	1853	1313	3424	6131	9970	14493
7c	1401567	31044	6024	2502	176	211	361	210	157	444	1491	1561	3395	6013	10165	10913
7e	1389314	31290	5940	2377	172	184	360	177	176	478	1303	1703	3370	6048	10296	9094
7g	1389556	31429	5766	2391	171	211	355	177	191	483	1232	1784	3346	5971	10300	9529
7i	1475505	33310	6145	2575	191	252	392	194	229	513	1523	1973	3595	6462	11029	12866
