# Supplementary material for: Phylogeny with introgression in Habronattus jumping spiders (Araneae: Salticidae)
Source: BMC Evol Biol. 2018 Feb 22;18:24. doi: 10.1186/s12862-018-1137-x (PMC5824460; doi:10.1186/s12862-018-1137-x)
Supplement: Supplementary file 3 — NEXUS file of the phylogenetic trees from various analyses (text, NEXUS format 250 kb) (TXT 244 kb) [file 12862_2018_1137_MOESM3_ESM.txt]

Table S3. D_{FOIL} results. Corresponding Figure indicated. Any p-values less than 0.0008 are considered significant (*, 0.05 level adjusted by a Bonferroni correction for 62 comparisons).

Fig.	Species 1	Species 2	Species 3	Species 4	Outgroup	D_{FO}	p (D_{FO})	D_{IL}	p (D_{IL})	D_{FI}	p (D_{FI})	D_{OL}	p (D_{OL})
***americanus* group**													
4b	*sansoni*	*americanus*	*ophrys*	*tarsalis*	*signatus*	0.232	*$7x10^{-12}$	0.230	*$1x10^{-11}$	-0.036	0.549	-0.043	0.5
***clypeatus* and *coecatus* groups**													
6a	*empyrus*	*pyrrithrix*	*clypeatus*	*aztecanus*	*ophrys*	0.072	0.036	0.037	0.29	-0.071	0.074	-0.12	0.0029
6c	*gilaensis*	*clypeatus*	*empyrus*	*pyrrithrix*	*ophrys*	-0.069	0.11	-0.137	0.0016	-0.0439	0.37	-0.132	0.0077
6e	*aztecanus*	*clypeatus*	*mexicanus*	*pyrrithrix*	*ophrys*	-0.160	*$5x10^{-9}$	-0.154	*$2x10^{-08}$	-0.025	0.429	-0.017	0.6
6g	*gilaensis*	*clypeatus*	*mexicanus*	*pyrrithrix*	*ophrys*	-0.104	0.001	-0.177	*$3x10^{-08}$	-0.024	0.627	-0.193	*$7x10^{-05}$
6i	*gilaensis*	*clypeatus*	*borealis*	*pyrrithrix*	*ophrys*	-0.014	0.7	-0.163	*$2x10^{-07}$	0.119	0.009	-0.189	*$3x10^{-05}$
6k	*aztecanus*	*clypeatus*	*borealis*	*pyrrithrix*	*ophrys*	-0.111	*$1.7x10^{-4}$	-0.152	*$2x10^{-07}$	-0.004	0.898	-0.053	0.097
**Deeper introgression**													
7a	*ophrys*	*aestus*	*zapotecanus*	*decorus*	*signatus*	-0.161	*$<10^{-14}$	-0.111	*$3x10^{-12}$	-0.010	0.688	0.116	*$4x10^{-06}$
7c	*ophrys*	*aestus*	*cambridgei*	*decorus*	*signatus*	-0.005	0.8	0.042	0.01	-0.003	0.899	0.110	*$1x10^{-05}$
7e	*ophrys*	*aestus*	*oregonensis*	*decorus*	*signatus*	0.104	*$2x10^{-10}$	0.111	*$1x10^{-11}$	0.068	0.008	0.085	$9x10^{-04}$
7g	*ophrys*	*aestus*	*jucundus*	*decorus*	*signatus*	0.140	*$<10^{-14}$	0.153	*$<10^{-14}$	0.064	0.010	0.097	*$1x10^{-04}$
7i	*ophrys*	*aestus*	*festus*	*decorus*	*signatus*	0.097	*$1x10^{-10}$	0.109	*$6x10^{-13}$	0.054	0.024	0.083	*$5x10^{-04}$
