# Supplementary material for: Phylogeny with introgression in Habronattus jumping spiders (Araneae: Salticidae)
Source: BMC Evol Biol. 2018 Feb 22;18:24. doi: 10.1186/s12862-018-1137-x (PMC5824460; doi:10.1186/s12862-018-1137-x)
Supplement: Supplementary file 4 — Concordance file for americanus group. (TXT 74 kb) [file 12862_2018_1137_MOESM4_ESM.txt]

Table S4. Patterson's D statistic results and counts of allele patterns. Corresponding figure indicated. Any p-values less than 0.0008 are considered significant (*, 0.05 level adjusted by a Bonferroni correction for 62 comparisons).

Fig.	Species 1	Species 2	D	p	ABBA	BABA	AAAA	AABA	ABAA	BAAA	BBAA	BBBA
***americanus* group** (Species 3 = *aestus*; outgroup = *signatus*)												
4d	*ophrys*	*tarsalis*	0.160	*$2x10^{-7}$	604	437	1460667	6699	2314	2323	2062	43518
	sansoni	*americanus*	0.039	$6x10^{-1}$	106	98	1314158	5972	763	465	2939	37177
	americanus	*tarsalis*	0.192	*$6x10^{-11}$	692	469	1234996	5253	2210	2500	1371	35308
	sansoni	*tarsalis*	0.165	*$7x10^{-9}$	717	514	1335239	5711	2439	2394	1427	38658
	sansoni	*ophrys*	0.0289	0.321	605	571	1546069	6759	2449	2340	1945	44248
	americanus	*ophrys*	0.0736	0.0166	569	491	1382876	5986	2132	2404	1780	38973
**VCCR clade** (Species 3 = *roberti*; outgroup = *ophrys*)												
5b	*gilaensis*	*jucundus*	-0.405	*$<10^{-14}$	514	1215	1100370	4025	4121	3201	1024	12356
5f	*festus*	*jucundus*	-0.177	*$<10^{-14}$	1031	1473	1315652	6191	5550	7773	1985	16703
5d	*festus*	*gilaensis*	0.377	*$<10^{-14}$	1051	476	1136547	4356	2939	5551	1541	13284
**Deeper introgression** (Species 3 = *ophrys*; outgroup = *signatus*)												
	zapotecanus	*decorus*	0.168	*$<10^{-14}$	2276	1620	1498519	10627	10931	16117	3547	34071
	cambridgei	*decorus*	-0.0040	$8x10^{-1}$	1868	1883	1466423	10183	11122	12084	3076	33903
	oregonensis	*decorus*	-0.114	*$4x10^{-12}$	1637	2058	1454931	10087	11288	10140	2970	34321
	jucundus	*decorus*	-0.164	*$<10^{-14}$	1537	2138	1449870	9819	11189	10558	2961	34214
	festus	*decorus*	-0.115	*$5x10^{-14}$	1906	2401	1551936	10659	12187	14479	3233	36798
